# Identification and Multidimensional Optimization of an Asymmetric Bispecific IgG Antibody Mimicking the Function of Factor VIII Cofactor Activity

**DOI:** 10.1371/journal.pone.0057479

**Published:** 2013-02-28

**Authors:** Zenjiro Sampei, Tomoyuki Igawa, Tetsuhiro Soeda, Yukiko Okuyama-Nishida, Chifumi Moriyama, Tetsuya Wakabayashi, Eriko Tanaka, Atsushi Muto, Tetsuo Kojima, Takehisa Kitazawa, Kazutaka Yoshihashi, Aya Harada, Miho Funaki, Kenta Haraya, Tatsuhiko Tachibana, Sachiyo Suzuki, Keiko Esaki, Yoshiaki Nabuchi, Kunihiro Hattori

**Affiliations:** Research Division, Chugai Pharmaceutical Co., Ltd., Gotemba, Shizuoka, Japan; Institut National de la Santé et de la Recherche Médicale, France

## Abstract

In hemophilia A, routine prophylaxis with exogenous factor VIII (FVIII) requires frequent intravenous injections and can lead to the development of anti-FVIII alloantibodies (FVIII inhibitors). To overcome these drawbacks, we screened asymmetric bispecific IgG antibodies to factor IXa (FIXa) and factor X (FX), mimicking the FVIII cofactor function. Since the therapeutic potential of the lead bispecific antibody was marginal, FVIII-mimetic activity was improved by modifying its binding properties to FIXa and FX, and the pharmacokinetics was improved by engineering the charge properties of the variable region. Difficulties in manufacturing the bispecific antibody were overcome by identifying a common light chain for the anti-FIXa and anti-FX heavy chains through framework/complementarity determining region shuffling, and by pI engineering of the two heavy chains to facilitate ion exchange chromatographic purification of the bispecific antibody from the mixture of byproducts. Engineering to overcome low solubility and deamidation was also performed. The multidimensionally optimized bispecific antibody hBS910 exhibited potent FVIII-mimetic activity in human FVIII-deficient plasma, and had a half-life of 3 weeks and high subcutaneous bioavailability in cynomolgus monkeys. Importantly, the activity of hBS910 was not affected by FVIII inhibitors, while anti-hBS910 antibodies did not inhibit FVIII activity, allowing the use of hBS910 without considering the development or presence of FVIII inhibitors. Furthermore, hBS910 could be purified on a large manufacturing scale and formulated into a subcutaneously injectable liquid formulation for clinical use. These features of hBS910 enable routine prophylaxis by subcutaneous delivery at a long dosing interval without considering the development or presence of FVIII inhibitors. We expect that hBS910 (investigational drug name: ACE910) will provide significant benefit for severe hemophilia A patients.

## Introduction

Hemophilia A is caused by an X-linked inherited dysfunction of coagulation factor VIII (FVIII). Patients with severe hemophilia A, who have plasma FVIII levels of less than 1% of normal, typically experience bleeding events several times a month [1]. Routine supplementation with exogenous human FVIII to maintain FVIII levels at 1% of normal or above is effective for reducing joint bleeding events and improving joint status and health-related quality of life in hemophilia A patients [2]. However, there are two major drawbacks to this prophylactic usage of exogenous FVIII. The first drawback is the necessity of frequent intravenous administration: three intravenous injections weekly of FVIII are necessary because of its low subcutaneous bioavailability and its short plasma half-life [3], [4], [5]. The second drawback is the development of inhibitory anti-FVIII alloantibodies, known as “inhibitors” [6]. Once FVIII inhibitors have developed, routine supplementation with exogenous FVIII will be no longer effective and the usage of exogenous FVIII for treating on-going bleeds is restricted. In such cases, alternative agents, such as activated factor VII and activated prothrombin complex concentrate, which are more expensive and have less stable hemostatic effects, need to be used to control bleeding [7], [8]. Therefore, a new agent that resolves these drawbacks of exogenous FVIII is awaited in the field of the bleeding prophylaxis of severe hemophilia A.

Monoclonal antibodies have become an important therapeutic option in numerous diseases and are expected to play a greater role in the future of disease treatment [9], [10]. Various monoclonal antibodies have been generated [11]; these not only include those with antagonistic activity but also those with agonistic activity [12], catalytic activity [13], and allosteric activity [14]. Antibody engineering technologies to generate bispecific antibodies have been extensively studied due to the huge potential of these antibodies for therapeutic applications [15]. Bispecific antibodies can be applied to simultaneously target two disease related antigens, retarget effector cells against the target cell [16], and co-ligate two different antigens on the same cell [17].

**Figure 1:**
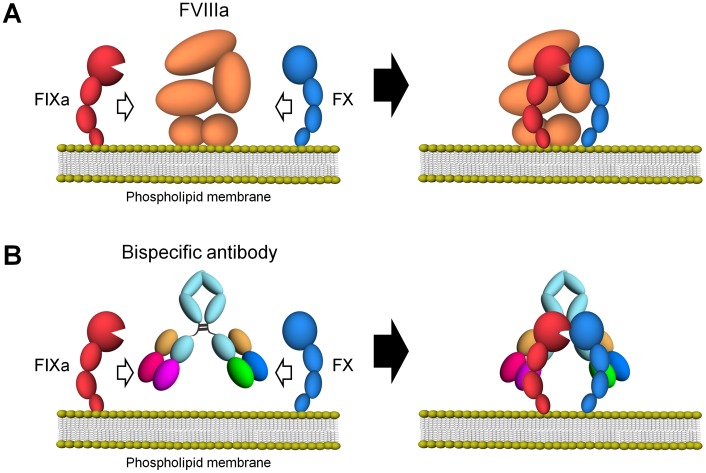
Schematic illustrations of cofactor actions of FVIIIa and a bispecific antibody promoting the interaction between FIXa and FX. (A) FVIIIa forms a complex with FIXa and supports the interaction between FIXa and FX through its binding ability to both factors on the phospholipid membrane. (B) A bispecific antibody binding to FIXa and FX would promote the interaction between FIXa and FX and exert FVIII-mimetic activity on the phospholipid membrane.

FVIII is cleaved by thrombin or factor Xa (FXa), and the resultant factor VIIIa (FVIIIa) presents a heterotrimeric structure consisting of the A1 subunit, the A2 subunit, and the light chain [18]. Simultaneous binding of FVIIIa to FIXa and FX by the light chain and the A2 subunit, and by the A1 subunit, respectively, contributes to FVIII cofactor activity which places FIXa and FX into proximity, and also allosterically enhances the catalytic rate constant of FIXa [19], [20], [21], [22], [23] (Fig. 1A).

Considering this function of FVIII and the versatilities of antibodies, we hypothesized that a bispecific antibody recognizing FIXa with one arm and FX with the other arm could mimic the FVIII cofactor activity by placing FIXa and FX in spatially appropriate positions, and by allosterically enhancing the catalytic activity of FIXa (Fig. 1B) [23]. We have recently reported a recombinant humanized bispecific antibody to FIXa and FX, termed hBS23, which exerted coagulation activity in FVIII-deficient plasma, even in the presence of FVIII inhibitors, and showed *in* *vivo* hemostatic activity in a cynomolgus monkey model of acquired hemophilia A, and had high subcutaneous bioavailability and a 2-week half-life in cynomolgus monkey [23]. Although the pharmacological concept of FVIII-mimetic bispecific antibody was clearly demonstrated in this report, the detail of this anti-FIXa/FX asymmetric bispecific IgG antibody identification was not described, and moreover, it required further optimization in several ways before the clinical use of such an agent in humans.

For therapeutic development, optimization of the bispecific antibody by molecular engineering to enable large-scale manufacturing of the bispecific antibody at clinical grade would be required. Although a variety of molecular formats for bispecific antibodies have been studied, including single-chain diabody, tandem scFv, IgG-scFv, DVD-Ig, CrossMab, and dual-binding Fab [24], we selected an asymmetric bispecific IgG format because it is the only format that can recognize FIXa and FX with each arm and has a long half-life and a native IgG structure. However, recombinant production of this format is challenging in comparison to the other formats because it consists of two different heavy chains and two different light chains which would result in the secretion of a mixture of ten different combinations of heavy and light chains [25]. Purification of one desired bispecific antibody from a mixture including nine miss-paired byproducts is nearly impossible. Engineering technologies to resolve such a difficulty have been previously reported. First, identification of a common light chain by phage display as the partner of the two heavy chains can reduce the number of heavy and light combinations to three: one heterodimeric bispecific antibody and two homodimeric monospecific antibodies [26]. However, selection of a common light chain with potent FVIII-mimetic activity based on the binding affinity by phage display was not feasible for our bispecific antibody, because higher binding affinities was considered not to lead to higher activity. To date, alternative approach to obtain common light chain has not been reported. Second, engineering the C_H_3 domain to facilitate Fc heterodimerization can minimize the amount of homodimeric byproducts [25], [26]. Nevertheless, the efficiency of heterodimerization is not complete; therefore, a small amount of homodimeric byproduct is formed, which needs to be removed in the downstream purification process. However, because the biophysical properties of the homodimeric byproducts are often similar to those of the target bispecific antibody, purifying the target bispecific antibody on a large scale for clinical applications is still challenging. To date, technologies to address this issue have not been reported. Therefore, improving the manufacturability of this type of FVIII-mimetic antibody by molecular engineering is required for therapeutic application.

Moreover, for maximizing the therapeutic potential of FVIII-mimetic bispecific antibody, optimization to increase the FVIII-mimetic activity of the bispecific antibody, prolong the half-life, improve the physicochemical properties of the antibody and reduce the immunogenicity of the humanized antibody would be required. This would enable more effective and long-term prophylaxis with stronger hemostatic effect for hemophilia A patients by a subcutaneous formulation with a longer dosing interval.

In this paper, we report molecular identification and multidimensional optimization of a FVIII-mimetic bispecific antibody which can be used for clinical application. At the start of this study, we examined bispecific combinations of large number of monoclonal anti-FIXa and anti-FX antibodies, and identified the lead anti-FIXa/FX bispecific IgG antibody having FVIII-mimetic activity. Then, this lead bispecific antibody was subjected to multidimensional optimization processes [27] to improve both its therapeutic potential and manufacturability. We successfully generated a humanized bispecific IgG antibody having sufficient FVIII-mimetic activity for prophylactic use even in the presence of FVIII inhibitors, high subcutaneous bioavailability with an approximately 3-week plasma half-life in cynomolgus monkeys, and minimal immunogenicity risk. In addition, molecular engineering enabled purification on a large manufacturing scale and formulation into a liquid formulation of 150 mg/mL for subcutaneous delivery. We expect that this anti-FIXa/FX bispecific antibody mimicking FVIII cofactor activity will provide significant benefit for managing bleeding events in severe hemophilia A patients.

## Results

### Research Flow of Identification and Multidimensional Optimization of Lead FVIII-mimetic anti-FIXa/FX Bispecific Antibody

**Figure 2:**
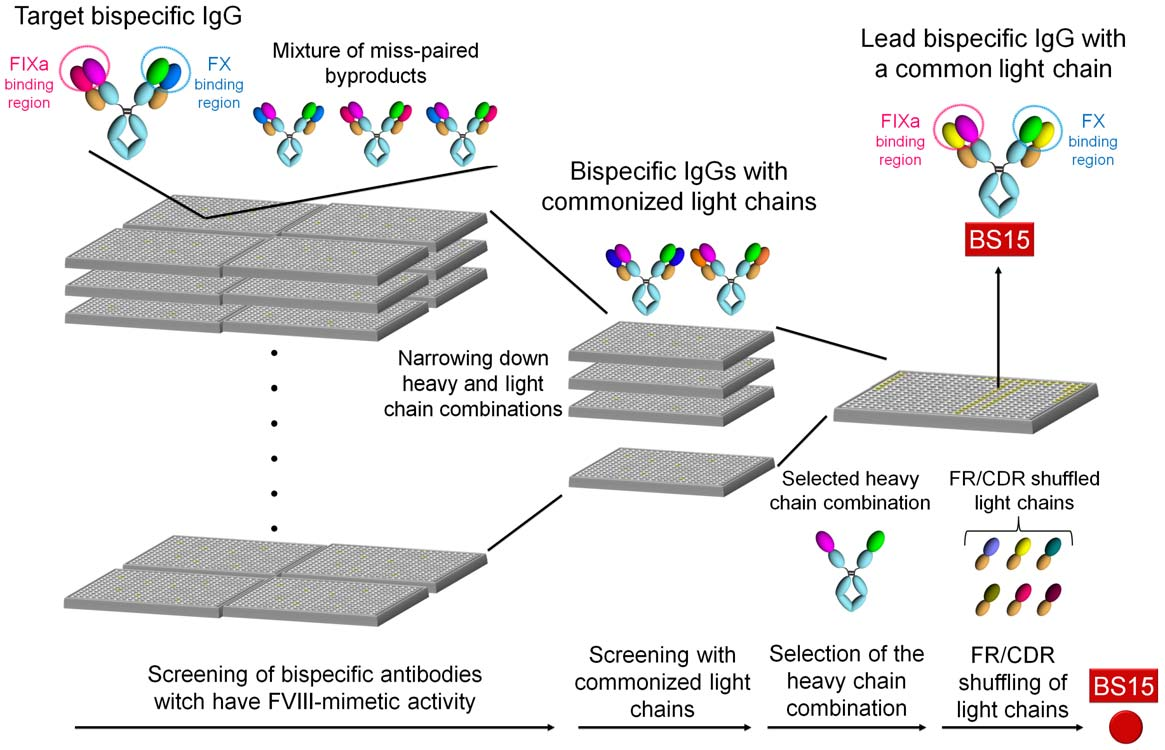
Flow of process to identify the lead bispecific antibody (BS15). BS15 was identified by combinatorial screening of bispecific antibodies, followed by screening of common light chains and then FR/CDR shuffling.

Figure 2 shows the flow of the screening process to identify the lead bispecific antibody with a common light chain (BS15). BS15 was generated by combinatorial screening of bispecific antibodies composed of anti-FIXa and anti-FX antibodies derived from immunization, followed by screening of common light chains and then framework/complementarity determining region (FR/CDR) shuffling.

**Figure 3:**
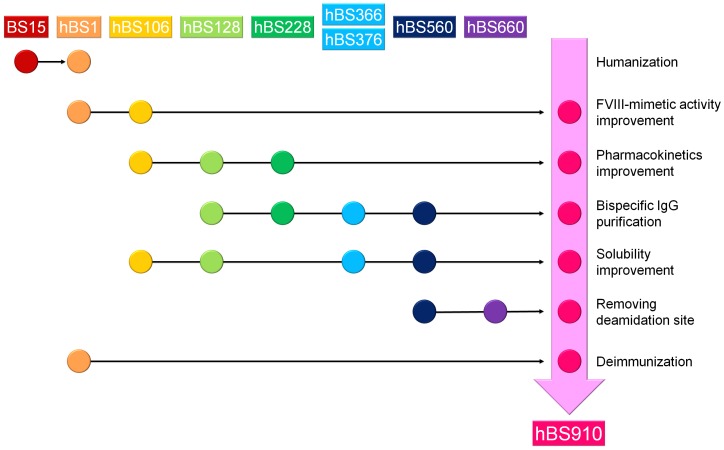
Multidimensional optimization flow to generate the bispecific antibody with most appropriate properties (hBS910). hBS910 was generated through multidimensional optimization with various antibody engineering technologies.

Figure 3 represents the multidimensional optimization flow to generate the bispecific antibody with the most appropriate properties for clinical application (hBS910) from the lead bispecific antibody (BS15). BS15 was firstly humanized to generate hBS1, followed by engineering to improve FVIII-mimetic activity (hBS106), improve pharmacokinetics (hBS128 and hBS228), enable purification of target bispecific antibody (hBS366 and hBS376), improve solubility (hBS560), remove deamidation site (hBS660), and reduce immunogenicity risk (deimmunization) to generate a multidimensionally optimized bispecific antibody (hBS910). Through this multidimensional optimization process, the numbers of variable region variants that we have generated for anti-FIXa heavy chain, anti-FX heavy chain and common light chain were approximately 500, 300 and 400, respectively, and the number of bispecific IgG antibodies that we have prepared and evaluated is approximately 2,400. Supplementary table S1 represents the number of mutations which were introduced into hBS1 to generate bispecific antibodies described in this report.

### Identification of Lead Anti-FIXa/FX Bispecific Antibody with FVIII-mimetic Activity

Approximately 200 monoclonal antibodies against FIXa or FX were obtained from animals immunized with either human FIXa or FX. Approximately 40,000 bispecific IgG antibodies that comprised different combinations of anti-FIXa and anti-FX antibodies in each arm were expressed. Although the expression product of two different heavy chain and two different light chain genes consists of a mixture of ten different species with different heavy and light chain combinations, including the one desired bispecific antibody and the nine miss-paired antibodies (miss-paired antibody includes two homodimeric antibodies with a correct heavy and light chain pair), Fc heterodimerization mutations would theoretically enable expression of an antibody mixture containing at least approximately 20% of the target bispecific antibody (see methods for detail) [25]. A total number of 94 bispecific antibodies, or combinations of anti-FIXa heavy chain and anti-FX heavy chain, which had FVIII-mimetic activity were successfully identified by an enzymatic assay. Heavy chain combinations were selected from the point of high FVIII-mimetic activity, not from the point of the similarity of the cognate light chain.

Next, in order to identify a common light chain for the different heavy chains to FIXa and FX, the selected heavy chain combinations were expressed with either one of cognate light chains. Out of 188 light chain commonized bispecific antibodies, the most potent one, termed c1, which consisted of rat anti-FIXa V_H_ and mouse anti-FX V_H_ chimerized with human IgG_4_ and rat anti-FIXa V_L_ chimerized with human κ (c1L), was selected for the next step.

Finally, in order to design a more potent common light chain, we performed FR/CDR shuffling. Since the cognate light chain for the selected anti-FX V_H_ (c2L) was not effective as a common light chain at all (data not shown), we tried to seek another effective common light chain with a high homology to c2L. From our antibody source, we identified a light chain (c3L) whose CDR sequence had >85% homology to that of c2L, and found out that it was effective as a common light chain for the selected two heavy chains. Therefore, we decided to use c3L for FR/CDR shuffling, too. Then, CDRs of c1L, c2L and c3L were shuffled among each other and grafted onto the FRs of c1L and c3L to generate twenty four light chain variants (supplementary Fig. S2A). Twenty four light chain variants were expressed with the two heavy chains, and the most potent common light chain, BS15L, a mouse–rat hybrid V_L_ chimerized with human κ, was identified (supplementary Fig. S2B). Thus, the light chain communized bispecific antibody with BS15L was selected as the lead chimeric bispecific IgG antibody (termed BS15).

### Humanization of Lead Chimeric Bispecific Antibody

The lead chimeric bispecific IgG antibody, BS15, was subjected to humanization. CDRs of the anti-FIXa heavy chain, the anti-FX heavy chain and the common light chain were grafted onto homologous human antibody FRs, which were the FRs of V_H_3, V_H_1 and V_κ_1 subfamilies respectively, by using a conventional CDR grafting approach [28]. A humanized bispecific IgG_4_ antibody, termed hBS1, was successfully generated while maintaining FVIII-mimetic activity (supplementary Fig. S3).

### Improving FVIII-mimetic Activity of the Bispecific Antibody

**Figure 4:**
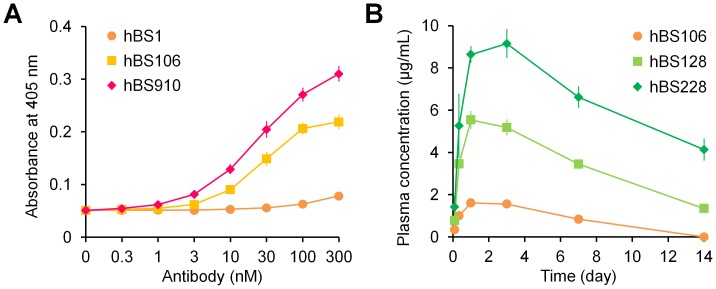
Improvement of therapeutic potential of the bispecific antibody. (A) Improving FVIII-mimetic activity of the bispecific antibody. Effect of hBS1 (circles), hBS106 (squares), and hBS910 (diamonds) on FX activation in the presence of FIXa, FX, and synthetic phospholipid is shown. The Y-axis indicates the absorbance at 405 nm of the chromogenic substrate assay (in many cases, the bars depicting s.d. are shorter than the height of the symbols). (B) Improving pharmacokinetics of the bispecific antibody. Time profiles of plasma concentration of hBS106 (circles), hBS128 (squares), and hBS228 (diamonds) in mice after subcutaneous injection at a dose of 1 mg/kg are shown. All the data were collected in triplicate and are expressed as mean ± s.d.

Although the humanized bispecific antibody hBS1 enhanced FX activation dose-dependently, demonstrating FVIII-mimetic activity, its therapeutic potential was marginal and its FVIII-mimetic activity needed to be improved for therapeutic application. Therefore, we explored mutations in the CDRs of hBS1 to improve the FVIII-mimetic activity, and we identified several effective mutations in the CDRs of the three chains. Following extensive studies to identify effective combinations of mutations that additively or synergistically improved FVIII-mimetic activity, we successfully generated hBS106. hBS106 demonstrated marked improvement of FVIII-mimetic activity over hBS1, including maximum activity, in the enzymatic assay (Fig. 4A).

During the course of subsequent optimization of hBS106 from the point of other aspects, FVIII-mimetic activity was monitored for each mutation so that the mutation would not compromise the activity. Moreover, further screening for mutations to further improve the activity was performed in parallel with other optimizations. Finally, we successfully generated hBS910, whose activity was even higher than that of hBS106 (Fig. 4A).

### Improving Pharmacokinetics of the Bispecific Antibody

To assess the pharmacokinetics of hBS106, the variant with improved FVIII-mimetic activity, the time course of the plasma concentration of this antibody (Fig. 4B) and its pharmacokinetic parameters (supplementary Table S2) were determined in mice. Clearance of hBS106 (67 mL/day/kg) was unexpectedly larger than the clearance of human IgG_4_ antibody reported in mice after subcutaneous injection (3–20 mL/day/kg) [29].

A homology model of hBS106 was used to explore the molecular features responsible for the poor pharmacokinetics, and a positive charge cluster was identified on the surface of Fv of the anti-FIXa arm (supplementary Fig. S4). To remove this cluster, we initially attempted subjecting lysine and arginine residues in the cluster to mutagenesis. However, these residues were found to be indispensable for the FVIII-mimetic activity (data not shown). Therefore, we explored the introduction of negatively charged residues near the cluster to neutralize the positive charge, and we identified a Tyr30Glu mutation in the common light chain that achieved this without any reduction in FVIII-mimetic activity. With hBS128, a variant of hBS106 with the single Tyr30Glu mutation, we observed improved plasma concentration and an approximately 4-fold improvement in the clearance compared to hBS106. Furthermore, the isoelectric point (pI) of hBS128 was lowered by introducing multiple mutations in the variable regions. hBS228, a variant of hBS128 with lowered pI, demonstrated further improved plasma concentration and approximately 2-fold improvement in clearance compared to hBS128 (Fig. 4B, supplementary Table S2). During the course of subsequent optimization of hBS228, we constantly made an effort to further improve the pharmacokinetics of the bispecific antibody.

### Isoelectric Point Engineering to Facilitate Purification of the Target Bispecific Antibody

**Figure 5:**
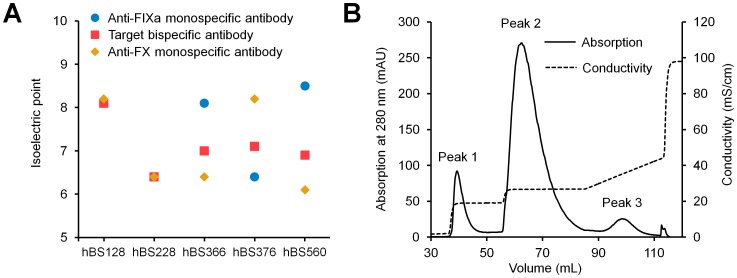
Isoelectric point engineering to facilitate purification of the target bispecific antibody. (A) Isoelectric points of target bispecific (squares) antibodies and homodimeric byproducts (anti-FIXa monospecific antibodies (circles) and anti-FX monospecific antibodies (diamonds)) determined by cIEF. (B) Cation exchange purification chromatogram of the target bispecific antibody of hBS560 from its homodimeric byproducts with step-wise elution with different NaCl concentrations. Peak 1, anti-FX homodimeric antibody; Peak 2, target bispecific antibody; Peak 3, anti-FIXa homodimeric antibody. Each peak area of peak 1, peak 2 and peak 3 was 9.9%, 85.7% and 4.4%, respectively.

Having a common light chain reduces the number of pairs of heavy and light chains to three, and engineering the C_H_3 domain enables preferential secretion of heterodimerized heavy chains. However, it is still difficult to completely prevent miss-paired homodimerization in large-scale production. Therefore, a downstream purification process to remove homodimeric byproducts is essential for pharmaceutical development. Ion exchange chromatography (IEC) is the major purification process by which to remove impurities after Protein A purification. The retention of IgG antibodies by IEC is determined by the electrostatic charge of the antibody molecule, which can be measured as its pI. Therefore, the pIs of hBS128 and hBS228, variants with improved pharmacokinetics, and pIs of their homodimeric byproducts were determined by cIEF (Fig. 5A). For both hBS128 and hBS228, the pIs of the bispecific antibody and the homodimeric byproducts were very close to each other, indicating that purification of the bispecific antibody hBS128 or hBS228 from the mixture of homodimeric byproducts is not feasible.

To facilitate the purification of the bispecific antibody, we implemented pI engineering into either one of the heavy chain variable regions to increase the pI difference between the bispecific antibody and the homodimeric byproducts. We successfully generated two variants, hBS366 (pI of the anti-FX heavy chain is lowered) and hBS376 (pI of the anti-FIXa heavy chain is lowered), in which FVIII-mimetic activity was maintained. The difference in the pI between the bispecific antibody and homodimeric byproducts markedly increased (Fig. 5A).

hBS366 was further optimized from the point of solubility (detail in the next paragraph), to generate hBS560. The target molecular form of hBS560 (heterodimeric bispecific antibody) was well separated from the two homodimeric byproducts by using cation exchange chromatography with step-wise elution (Fig. 5B). During the course of subsequent optimization of hBS560, the pI difference between the two heavy chains was carefully maintained. The multidimensionally optimized variant hBS910 was capable of being purified on a large production scale (2500-liter fermentation).

### Improving Solubility Properties of the Bispecific Antibody

**Figure 6:**
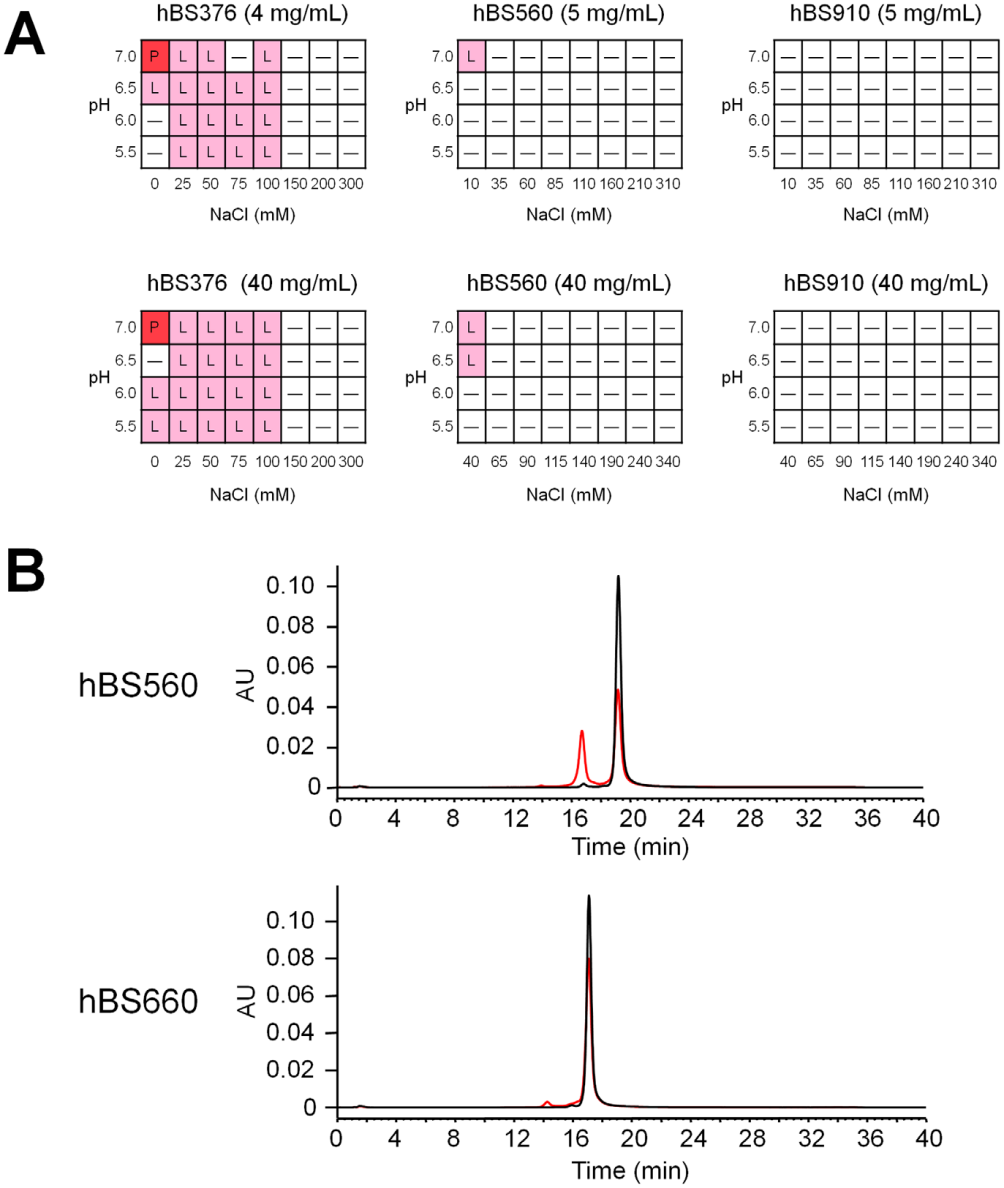
Improvement of pharmaceutical properties of bispecific antibodies. (A) Antibody solution profiles of hBS376 and hBS560 at different antibody concentrations, pH, and NaCl concentrations. The antibody solution under each condition was photographed and the state determined (P, precipitation; L, liquid–liquid phase separation; –, clear liquid). (B) Cation exchange chromatography of hBS560 and hBS660 before (black) and after incubation at 40°C for 2 weeks (red). Acidic peak indicating deamidation at HCDR3 increased after incubation at 40°C for 2 weeks for hBS560. No marked increase of acidic peak was observed for hBS660.

hBS106, a variant with improved FVIII-mimetic activity, had unexpectedly low solubility, exhibiting either precipitation or liquid–liquid phase separation [30] (supplementary Fig. S5). This lack of solubility was partially due to the positive charge cluster, since the Tyr30Glu mutation described above markedly improved the solubility. However, for hBS376, the variant whose anti-FIXa heavy chain pI was lowered, at concentrations of 4 and 40 mg/mL, precipitation and phase separation still occurred in phosphate buffer of pH 5.5 to 7.0 and NaCl of 100 mM or less (Fig. 6A).

To improve the solubility of hBS376, mutations in the variable regions of hBS376 were explored. Several effective mutations including substitutions of hydrophobic residues into hydrophilic residues were identified, and their combinations successfully generated hBS560. Precipitation and phase separation of hBS560, the variant with improved solubility, was markedly suppressed and occurred only below 40 mM NaCl (Fig. 6A). The solubility of hBS560 was more than 100 mg/mL. During the course of subsequent optimization of hBS560, the effect of mutations on the solubility was constantly monitored and we made further efforts on improving the solubility. We successfully generated hBS910, a multidimensionally optimized variant, which did not exhibit precipitation or phase separation under the conditions tested (Fig. 6A). Furthermore, hBS910 could be concentrated up to at least 200 mg/mL.

### Removing Deamidation Site in the CDR of the Bispecific Antibody

An accelerated stability study revealed that hBS560 exhibited asparagine deamidation in the third complementarity-determining region of the heavy chain (HCDR3) (Asn99) of the anti-FIXa arm after incubation at 40°C for 2 weeks, as shown by the increase in the acidic peak in the cation exchange chromatography analysis (Fig. 6B) [31], and reduction of FVIII-mimetic activity was observed (data not shown). A single mutation of Asn99 to another amino acid to remove this deamidation site was not feasible due to the loss of FVIII-mimetic activity and solubility. Subsequently, a double mutation was explored, and hBS660, in which a His98Arg and Asn99Glu double mutation was introduced to hBS560, was identified to maintain the FVIII-mimetic activity. hBS660 showed no increase in the acidic peak after incubation, demonstrating that the deamidation site was removed (Fig. 6B). During the course of subsequent optimization of hBS660, deamidation was carefully monitored, and successfully generated hBS910, a multidimensionally optimized variant, which did not exhibit deamidation.

### Deimmunization of Humanized Bispecific Antibody by Removing T-cell Epitopes

During the course of multidimensional optimization of hBS1, the effects of each mutation on immunogenicity were evaluated by Epibase (Lonza), an *in silico* T-cell epitope prediction system [32]. Any mutation that was predicted to increase the potential immunogenicity risk was avoided as much as possible. Simultaneously, to generate a bispecific antibody with minimum immunogenicity risk, any mutation that was predicted to decrease the potential immunogenicity risk by reducing the number of T-cell epitopes was screened.

The immunogenicity risk score of hBS910, the multidimensionally optimized variant, was markedly decreased compared to the lead chimeric antibody (BS15) and its humanized version (hBS1), and was comparable to trastuzumab and palivizumab which are non-immunogenic in clinical (supplementary Fig. S6A). Moreover, EpiMatrix (EpiVax), another *in silico* immunogenicity prediction system [33], [34], also predicted that the sequence of hBS910 was minimally immunogenic (supplementary Fig. S6B).

### Therapeutic Potential of the Multidimensionally Optimized Bispecific Antibody, hBS910

**Figure 7:**
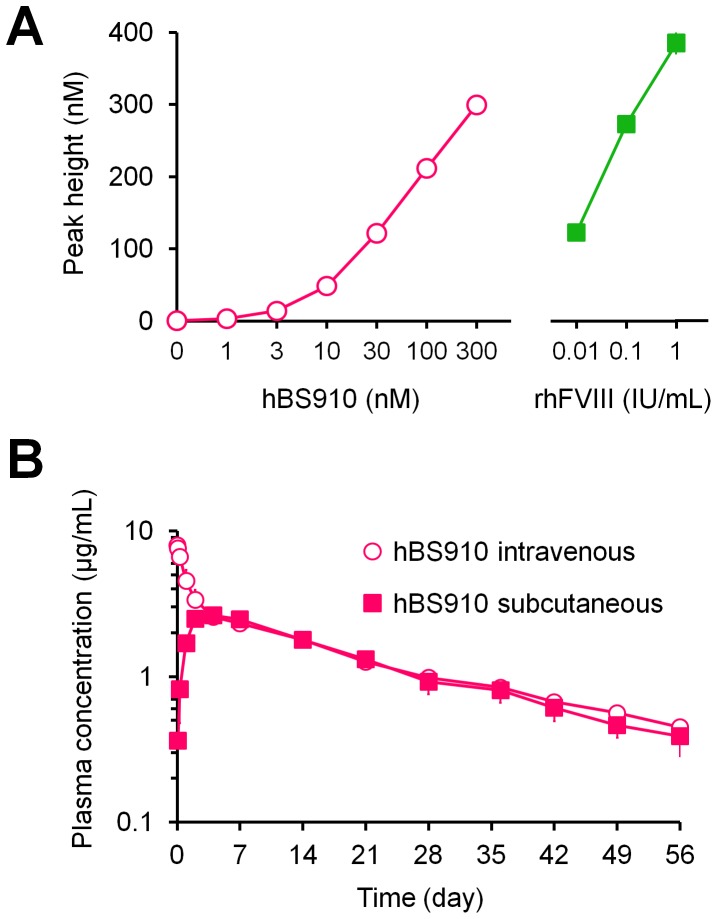
Therapeutic potential of multidimensionally optimized bispecific antibody, hBS910. (A) FVIII-mimetic activity of hBS910 in thrombin generation assay (TGA). Effect of hBS910 (circles) or recombinant human FVIII (squares) on thrombin generation in FVIII-deficient plasma is shown. The reaction was triggered by FXIa, synthetic phospholipid, and Ca^2+^. The Y-axis indicates the peak height, a thrombin generation parameter (in many cases, the bars depicting s.d. are shorter than the height of the symbols). Data were collected in triplicate for each plasma lot and are expressed as mean ± s.d. (B) Pharmacokinetics of hBS910 in cynomolgus monkeys. Time profiles of plasma concentration of hBS910 after intravenous (circles) or subcutaneous (squares) injection are shown.

To examine the therapeutic potential of hBS910, its FVIII-mimetic activity was compared with human FVIII by using thrombin generation assay (TGA) [23], [35], [36] in commercially available human FVIII-deficient plasma which was derived from a single donor with severe hemophilia A without FVIII inhibitors (Fig. 7A). hBS910 dose-dependently increased peak height (defined as the peak concentration of free thrombin) in the same manner as recombinant human FVIII (rhFVIII). The thrombin generation activity of hBS910 was also observed even in plasma of a hemophilia A donor who has FVIII inhibitors, whereas 1 IU/mL of rhFVIII did not exhibit any effects (data not shown). On the other hand, while polyclonal anti-idiotype antibodies to the anti-FIXa Fab or anti-FX Fab of hBS910 completely inhibited the activity of hBS910, they did not interfere with rhFVIII activity at all (data not shown).

To assess the potential for subcutaneous delivery with a long dosing interval, hBS910 was intravenously and subcutaneously administrated to cynomolgus monkeys, and the time course of the antibody plasma concentration and pharmacokinetic parameters were obtained (Fig. 7B, supplementary Table S3). The subcutaneous bioavailability was sufficiently high (86%) and the plasma half-life was approximately 3 weeks. Moreover, hBS910 could be formulated into a 150 mg/mL liquid formulation for subcutaneous delivery in the clinical setting without any significant aggregation or degradation during storage.

## Discussion

The lead bispecific antibody was identified from approximately 40,000 different bispecific antibodies. Bispecific antibodies meeting the criteria for FVIII cofactor activity were extremely rare (<0.3%). This seems reasonable since such a bispecific antibody requires simultaneous binding to the appropriate epitope of both FIXa and FX in order to place these two factors into a spatially appropriate position and precisely bring the catalytic site of FIXa close to the cleavage site of FX. Requirement of simultaneous binding to FIXa and FX by a single bispecific antibody was supported by the fact that only a bispecific antibody, and neither monospecific antibodies nor a mixture of them, exhibited FVIII-mimetic activity (supplementary Fig. S7).

Generally, the biological activity of antagonistic antibodies can be improved by increasing the binding affinity to the target antigen [37], [38]. The biological activity of agonistic antibodies, on the other hand, is reported to be inversely correlated with the affinity to the antigen, presumably due to the necessity to dissociate from the antigen to repeatedly induce agonistic signals to the target [39]. In the case of our bispecific antibody, the antibody needs to bind to both FIXa and FX with sufficient affinity to promote the interaction between the factors, while after FX activation by FIXa, FXa needs to be rapidly dissociated from the antibody to proceed to the subsequent coagulation reaction and to enable the antibody to turn over. Therefore, we assumed that a simple increase in the binding affinity to both FIXa and FX would not necessarily improve the FVIII-mimetic activity. Consequently, generation of variants that had improved activity required screening of a large number of variants in which mutations had been introduced via structure-based or random mutagenesis. These mutations were mainly introduced to the residues that were predicted to directly or indirectly contact the antigen by the homology modeling of the lead antibody or experimentally identified to affect FVIII-mimetic activity.

To gain insights into the mechanisms underlying the improvement in activity, kinetic analyses of hBS1, hBS106, and hBS910 (bispecific antibodies each with different FVIII-mimetic activity) binding to FIXa and FX were performed by SPR analysis (supplementary Fig. S8A, B). However, because the kinetic parameters of these antibodies were differently affected by the fitting conditions of the sensorgrams, we were not able to obtain meaningful kinetic parameters with which to compare these antibodies. Nevertheless, the binding properties of these antibodies were obviously different, and these changes might have contributed to the improvement in activity. It appears that the binding affinity of hBS910 to FIXa is weaker than hBS1 and hBS106, and hBS910 has faster association and dissociation rate for FX. Although this tendency might result in rapid turnover of FX activation by the bispecific antibody and explain the highest FVIII-mimetic activity of hBS910, it does not explain the large difference of FVIII-mimetic activity between hBS1 and hBS106. Since bispecific antibody needs to strictly place catalytic site of FIXa to the cleavage site of FX and may require allosteric effect on FIXa in order to mimic the function of FVIII, it can be postulated that the improved FVIII-mimetic activity of hBS106 compared to hBS1 may be derived not only from the changes in the binding kinetics but also from the subtle changes in the binding epitope, binding angle or allosteric effect caused by the mutations introduced.

The FVIII-mimetic activity of 30 nM hBS910 was equivalent to rhFVIII activity at 0.01 IU/mL (1% of normal level), and the activity of 300 nM hBS910 was greater than rhFVIII activity at 0.1 IU/mL (10% of normal level) in the TGA using human FVIII-deficient plasma. The activity was also observed even in the presence of FVIII inhibitors, which is reasonable considering the lack of homology between the sequences of hBS910 and FVIII. Recently, we have demonstrated that FVIII-mimetic bispecific antibody, termed hBS23, exerts hemostatic activity *in* *vivo* in acquired hemophilia A model using cynomolgus monkey, which was considered sufficient for routine prophylaxis [23]. hBS23 is one of the FVIII-mimetic activity improved variants obtained during the multidimensional optimization to generate hBS910, and has similar FVIII-mimetic activity as hBS106. Kinetic analysis [23] of hBS23 and hBS910 demonstrated that hBS910 showed two times the effect on increasing *k*_cat_ */K*_m_ compared to hBS23 (unpublished data). In human FVIII-deficient plasma, hBS910 required only two third of plasma concentration to exhibit the equivalent activity to hBS23 in the TGA, suggesting that hBS910 has 1.5 times the activity to promote thrombin burst (unpublished data). When compared with FVIII, this *in* *vivo* hemostatic effect was consistent with its *in* *vitro* activity in TGA. Thus, the above results clearly demonstrate that hBS910, which is more potent than hBS23, could exert FVIII-mimetic activity in hemophilia A patients sufficient to achieve routine prophylaxis regardless of the presence of FVIII inhibitors. The amino acid sequence of hBS23 is described in our patent as an antibody name Q153-G4k/J142-G4h/L180-k, and that of hBS910 is described in the patent [Igawa T, Sampei Z, Kojima T, Soeda T, Muto A, et al. Multi-specific antigen-binding molecule having alternative function to function of blood coagulation factor VIII. WO/2012/067176].

It is highly desirable that our bispecific antibody be able to be administered subcutaneously with a long interval between doses. The poor pharmacokinetics of hBS106 in mice was partially attributed to the large positive charge cluster, and the single Tyr30Glu mutation to neutralize this charge cluster markedly improved the pharmacokinetics. Such a charge cluster may increase non-specific binding to the extracellular matrix which would increase the clearance of the molecule. Recently, it was reported that antibodies with lower pI have better pharmacokinetics, and engineering antibodies to lower the pI improved their pharmacokinetics [29]. This approach was successfully applied to our bispecific antibody. hBS910 exhibited a plasma half-life of approximately 3 weeks in cynomolgus monkeys, which is longer than the half-lives of hBS23 (approximately 2 weeks) [23] and other humanized or fully human IgG antibodies [40], [41], presumably due to the benefit of pI engineering. Since the half-lives of IgG antibodies are generally longer in humans than in cynomolgus monkeys [42], we expect that hBS910 would have a half-life of at least 3 weeks in humans, which is overwhelmingly longer than that of exogenous FVIII (0.5 days) [4]. Furthermore, hBS910 exhibited 86% subcutaneous bioavailability, consistent with that of other IgG antibodies [24], [43], [44], providing a huge advantage over exogenous FVIII which requires intravenous administration [5]. This high subcutaneous bioavailability and long half-life of hBS910 strongly supports the feasibility of routine prophylaxis by subcutaneous administration with a long dosing interval.

We utilized an *in silico* T-cell epitope prediction system, Epibase, to minimize the number of T-cell epitopes present in the bispecific antibody. The immunogenicity risk of hBS910 was predicted not only by Epibase but also by EpiMatrix to be comparable with that of non-immunogenic antibodies. Considering that up to 30% of patients develop inhibitors against exogenous FVIII [45], immunogenicity is an important issue in the routine prophylaxis of hemophilia A. Although the true immunogenicity of hBS910 needs to be evaluated clinically, it is noteworthy that two different *in silico* systems predicted hBS910 to be non-immunogenic. However, the possibility that a small number of patients could develop anti-hBS910 antibodies cannot be ruled out. In case hBS910 becomes ineffective owing to development of anti-hBS910 antibodies, it is important that they do not cross-react with FVIII so that exogenous FVIII treatment remains as an alternative. In addition to the fact that there is no homology between the amino acid sequences of hBS910 and FVIII, the risk of such cross-reactivity was found to be negligible because polyclonal anti-idiotype antibodies against hBS910 did not inhibit the thrombin generation activity of FVIII. This demonstrates that the development of anti-hBS910 antibodies would not compromise the use of exogenous human FVIII therapy.

No recombinant bispecific IgG antibody has yet reached the market. One of the reasons is the difficulty in large-scale manufacturing at clinical grade. Identification of a common light chain is an important step for manufacturing asymmetric bispecific IgG antibodies. A previous study utilized a phage display library to identify a common light chain for the two heavy chains against different antigens [26]. However, since high-affinity binding to FIXa or FX would not necessarily result in high FVIII-mimetic activity, selection of a common light chain with potent activity based on the binding affinity by phage display was not feasible. We successfully identified a potent common light chain by a novel FR/CDR shuffling approach. Since we were able to obtain the common light chain (BS15L) that had much better potency than the parental light chain (c1L) from the initial twenty four light chain variants, we did not perform further shuffling of FRs. Because residues in the FR often affects antigen binding of the antibody, we propose that shuffling of both CDRs and FRs would be an efficient and general approach to identify potent common light chains for bispecific antibodies. This novel approach enables incorporation of the beneficial residues from each CDR and FR into a common light chain. Although it might be not suitable for screening common light chain against large panels of heavy chains, this approach can be generally applicable for identification of a common light chain for the selected pair of heavy chains without using a library display system. In our other asymmetric bispecific antibodies with the same molecular format, common light chains for two different heavy chains could be successfully identified by this approach. In some cases, it was possible to directly identify humanized common light chain by performing CDR shuffling on the human FRs. This could be a more efficient approach since it abbreviates the following humanization process. However, since FR residues are often important for antigen binding property, we took more cautious approach in this FVIII-mimetic bispecific antibody, and performed CDR shuffling on its cognate FRs to identify a potent common light chain, followed by humanization of the identified common light chain.

Although the heavy chain heterodimerization efficiency depended on the expression balance of each heavy chain, our Fc heterodimerization mutations achieved approximately 85% efficiency for hBS560 in the system of unoptimized expression balance (Fig. 5B). Nevertheless, expression of a small amount of monospecific homodimeric antibodies is inevitable even with Fc heterodimerization mutations. In the case of our bispecific antibody, byproducts are anti-FIXa and anti-FX bivalent monospecific antibodies. These byproducts are not simply impurities with no activity, but they have the potential to competitively inhibit the activity of the bispecific antibody by bivalent binding to the factors. Therefore, homodimeric byproducts need to be removed as much as possible by a downstream process. However, the only molecular difference between a homodimeric byproduct and the bispecific antibody is the heavy chain variable region of one arm. Since antibody variable regions generally have similar sequences except for the CDRs, it was assumed that separation of such byproducts from the bispecific antibody by IEC would be difficult. Therefore, we took the advantage of pI engineering, and engineered the heavy chain variable region to increase the pI difference between the bispecific antibody and the byproducts, thereby improving the separation by IEC. pI engineered hBS910 could be actually purified from 2500-liter fermentation by Protein A and IEC using conventional antibody purification processes. Such a novel pI engineering approach would be generally applicable to facilitate the purification of bispecific IgG antibodies.

To realize subcutaneous delivery in a clinical setting, bispecific antibodies need to be formulated into a high concentration (i.e. >100 mg/mL) since the volume that can be subcutaneously injected is generally limited to less than 1.5 mL [46]. However, hBS376 exhibited phase separation even at 4 mg/mL. Phase separation of antibody solutions into an upper phase with low antibody concentration and a lower phase with high antibody concentration not only precludes high concentration formulation but also makes downstream purification processes difficult [30]. It has been recently reported that low solubility of antibodies has been overcome by introducing specific mutations into the molecular surface [47], but mutations to prevent phase separation have not been reported. We demonstrated that phase separation of hBS376 could also be eliminated by introducing multiple mutations into the molecular surface. hBS910 exhibited no phase separation under the conditions tested, and could be concentrated up to at least 200 mg/mL without any issue.

Since FVIII is unstable under liquid formulation, all the marketed FVIII agents are distributed as lyophilized formulation, and therefore require reconstitution before injection. Liquid formulation allows injection without this process, and thus would be much more convenient for the patients. However, monoclonal antibodies stored in aqueous solution often undergo deamidation of the asparagine residues in the CDRs resulting in reduction of the biological activity of the antibody, which was the case for hBS560 [31]. Although the general strategy is to remove the deamidation site by mutating the asparagine residue itself to another amino acid, this strategy was not feasible in the case of hBS560. A simultaneous double mutation approach enabled storage of hBS910 at 40°C for 2 weeks without reduction of activity. Consequently, hBS910 could be stably stored in a patient-friendly 150 mg/mL liquid formulation, which would enable approximately 3 mg/kg subcutaneous delivery in humans (1.5 mL injection for a 75 kg patient). Considering the FVIII-mimetic activity determined by TGA and the pharmacokinetics in cynomolgus monkeys, such a formulation would provide effective prophylaxis by subcutaneous delivery with a long dosing interval.

In conclusion, we have generated a novel humanized anti-FIXa/FX bispecific IgG antibody, hBS910, through a process of identifying the lead candidate from approximately 40,000 bispecific combinations, followed by a multidimensional optimization process to improve both the therapeutic potential and the manufacturability. hBS910 overcomes the two major drawbacks of routine prophylaxis by exogenous FVIII. First, while exogenous FVIII requires frequent intravenous administration, hBS910 can be subcutaneously administered with a long dosing interval. Second, while the development of FVIII inhibitors is a critical issue for exogenous FVIII, our study suggests that hBS910 can be used without fear of developing FVIII inhibitors and can be used in patients who have already developed FVIII inhibitors. We believe that hBS910, with its multidimensionally optimized profile, will provide significant improvement in the quality of life of hemophilia A patients by reducing not only bleeding but also the burden on the patients themselves, their parents, and all medical staff. Potential of hBS910 (investigational drug name; ACE910) in hemophilia A patient is currently being evaluated in clinical study.

## Materials and Methods

### Ethics Statement

Animal studies were performed in accordance with the Guidelines for the Care and Use of Laboratory Animals at Chugai Pharmaceutical Co., Ltd. under the approval of the company’s Institutional Animal Care and Use Committee. The company is fully accredited by the Association for Assessment and Accreditation of Laboratory Animal Care International (http://www.aaalac.org). Details of cynomolgus monkey care and maintenance, including shelter and availability of food, water, and environmental enrichment are as follows. Identification of individuals; individual animals were identified by microchip number. Each cage was identified by a cage number indicated on the cage rack, cage type; a stainless steel cage, housing density; 1 animal/cage, temperature; 18°C to 28°C, relative humidity; 35% to 75%, air change frequency; at least 10 times per h, illumination timing; 12 h per day, from 7∶00 am to 7∶00 pm, feed; cynomolgus monkeys were daily provided approximately 100 g of solid chow (Certified Primate Diet 5048, Japan SLC) and supplementary foods (1/2 peeled banana or 50 g of sweet potato) and drinking water; animal room tap water, provided ad libitum using the automatic water supply system. The studies involved injection of the agents and collection of blood samples which did not require procedures that would cause more than slight or momentary pain or distress to the animals. All injections and blood collection were conducted by trained and qualified primate therapeutic staff in Chugai Pharmaceutical. Provisions were made in the approved protocol for veterinary intervention in the case of any distress or morbidity from the injected agent; however no adverse events occurred during the studies or during the recovery period. Therefore, no scarification of the animals was conducted.

### Generation of Anti-FIXa/FX Bispecific Antibodies

Approximately 200 monoclonal antibodies to FIXa or FX were obtained from FIXa or FX immunized mice, rats, and rabbits. The V_H_ and V_L_ of those antibodies were then combined with engineered human IgG_2_ or IgG_4_ that included mutations to facilitate Fc heterodimerization. These engineered human IgG_2_ or IgG_4_, which included the knobs-into-holes mutations [26], were generated by introducing the same substitutions as those of IgG_1_ knobs-into-holes to the corresponding positions of IgG_2_ and IgG_4_ heavy chains, and were used through the screening for the lead identification. Anti-FIXa/FX bispecific were generated by HEK293 cells co-transfected with mixture of four plasmids encoding anti-FIXa heavy and light chain and anti-FX heavy and light chain (supplementary Fig. S1). At the screening step for lead identification, we used knobs-into-holes mutations which could achieve at least 90% efficiency of the two heavy chain heterodimerization. If the assembly of a heavy chain with two light chains occurs with equal probability, 25% of heavy chain heterodimeric antibodies will have the correct heavy and light chain pair [25]. Theoretically, in the supernatant of transfected cells, this would result in at least approximately 20% (90%×25% = 22.5%) of antibodies to be the target bispecific antibody, and less than 3% (10%×25% = 2.5%) of antibodies to be homodimeric antibodies with a correct heavy and light chain pair. Transfected cells were cultured in 96-well culture plates, and either filtrated culture supernatants or Protein A purified antibodies were used for evaluation. We also generated bispecific antibodies with a common light chain, anti-FIXa monospecific IgG consisting of anti-FIXa heavy chain and a common light chain, and anti-FX monospecific IgG consisting of anti-FX heavy chain and a common light chain by the method described above. The engineered human IgG_4_ including the knobs-into-holes mutations was used during the early stage of the multidimensional optimization, and a different engineered IgG_4_ including electrostatic steering mutations [25] was used during the late stage optimization. By using the engineered IgG_4_, approximately 85–95% of the Protein A purified antibody would be the target bispecific antibody and the rest would be homodimeric antibodies. For the detailed characterization of hBS910, homodimeric antibodies were removed by ion exchange chromatography.

### Screening Bispecific Antibodies for FVIII-mimetic Activity (Enzymatic Assay)

The ability of each antibody to enhance FIXa-catalized FXa generation was evaluated in an enzymatic assay with purified human coagulation factors (Enzyme Research Laboratories). The FXa generation reaction was performed in the presence of 1 nM human FIXa, 140 nM human FX, 20 µM synthetic phospholipid (10% phosphatidylserine, 60% phosphatidylcholine and 30% phosphatidylethanolamine; Avanti Polar Lipids) prepared as previously described [48], and antibodies at room temperature for 2 min in TBS containing 5 mM CaCl_2,_ 1 mM MgCl_2_ and 0.1% (wt/vol) BSA (pH 7.6). The reaction was stopped by the addition of EDTA at appropriate time points. The activity of the FXa generated was determined by absorbance at 405 nm after the addition of chromogenic substrate S-2222 (Chromogenix). Data were collected in triplicate.

### FR/CDR Shuffling of the Light Chains

CDRs of three light chains (c1L, c2L and c3L) were shuffled among each other then grafted onto the FRs of either c1L or c3L to generate light chain variants (supplementary Fig. S2A). Since c2L and c3L had identical CDR1 and CDR2 sequences, we generated twenty four light chains (twelve CDR combinations in two FRs) including parental c1L and c3L. Light chain variant genes were generated by assembly PCR, and the twenty four bispecific antibodies with these light chains were prepared as described above. FVIII-mimetic activity of each antibody was evaluated in APTT assay with standard techniques using APTT reagent (Sysmex) and FVIII deficient plasma (Sysmex).

### Pharmacokinetic Study of Bispecific Antibodies in Mice

1 mg/kg doses of each bispecific antibody were administered to C57BL/6J normal mice (Charles River) by single subcutaneous injection (*n* = 3 for each group). Blood samples were collected at an appropriate time after each administration. Plasma concentration of bispecific antibodies was determined by human IgG-specific ELISA. Pharmacokinetic parameters were calculated by WinNonlin Professional software (Pharsight).

### Determination of Isoelectric Point (pI) by Capillary Isoelectric Focusing (cIEF)

cIEF analyses of antibodies were performed with a PA800 plus Pharmaceutical Analysis System and 32 Karat software (Beckman Coulter) as described in the cIEF application guide (PN A78788AA). Briefly, antibody solutions (approximately 1 mg/mL in PBS) were diluted 1∶25 with cIEF master mix solution containing cIEF gel, urea, Pharmalyte 3–10, arginine, iminodiacetic acid, and pI markers. A neutral capillary was preconditioned by rinsing with urea solution, and samples were injected and focused. The pI of each of the antibodies was determined from the pI markers.

### Separation of Bispecific Antibodies by Cation Exchange Chromatography

Using an AKTAexplorer 10S (GE Healthcare), two HiTrap SP FF 1 mL columns (GE Healthcare) were connected in tandem and equilibrated by 20 mM sodium phosphate, pH 6.0. The elution buffer contained an appropriate concentration of NaCl in this equilibration buffer. The load sample was first prepared from culture supernatant using MabSelect Sure (GE Healthcare), and was then dialyzed with equilibration buffer. The sample antibody solution was applied to the HiTrap SP FF column at 1.5 mg/mL resin. After washing with equilibration buffer, antibodies were eluted with 10 column volumes (CV) of equilibration buffer containing 150 mM NaCl and then with 15 CV of 220 mM NaCl buffer in a stepwise manner, and finally with 15 CV of NaCl buffer in a linear gradient to 450 mM.

### Solubility Analysis of Bispecific Antibodies

Stock solutions of bispecific antibodies were prepared by dialysis against water or 50 mM NaCl solutions, followed by ultrafiltration concentration. Samples were prepared by adding formulation stock solution to antibody stock solution using a Hydra II Plus One liquid-handling robot (Matrix). After centrifugation, 1 µL of each sample was stored in an Intelli-Plate 96-2 (Art Robbins) at 20°C for 1 day, and then images of each well were taken by Rock Imager 54 (Formulatrix). The state of each antibody solution was determined as either a clear solution, precipitation, or liquid–liquid phase separation.

### Accelerated Stability Study of Bispecific Antibodies

Solutions of bispecific antibodies were dialyzed against PBS, pH 7.4 (Sigma). 1 mg/mL of each antibody solution was stored at 40°C for 2 weeks and then analyzed by cation exchange chromatography (IEC) using BioPro SP columns (YMC) at room temperature. The mobile phase (A) was 20 mM sodium phosphate, pH 5.8, and the mobile phase (B) was 20 mM sodium phosphate and 500 mM NaCl, pH 5.8.

### *In silico* Evaluation of Immunogenicity of the Variable Region of Bispecific Antibodies

T-cell epitope prediction of the bispecific antibody variants and immunogenicity risk scores of antibodies were provided by Epibase (Lonza). Immunogenicity scales of antibodies were provided by EpiMatrix (EpiVax).

### Thrombin Generation Assay (TGA)

Calibrated automated thrombography [35] was employed using a 96-well plate fluorometer (Thermo Fisher Scientific) equipped with a 390/460 filter set, a dispenser, and the analyzing software (Thrombinoscope software version 3.0.0.29; Thrombinoscope) to measure thrombograms. Briefly, each concentration of bispecific antibody or rhFVIII (Bayer Healthcare) was added to FVIII-deficient plasma (<1% FVIII activity) either without inhibitors or with inhibitors against FVIII (George King Bio-Medical). Each concentration of bispecific antibody or rhFVIII was also added to plasma containing polyclonal rabbit anti-idiotype antibodies against anti-FIXa Fab or anti-FX Fab (300 µg/mL each). Into each well was dispensed 80 µL of the plasma, to which was then added 20 µL of the triggering solution containing 0.47 nM human FXIa (Enzyme Research Laboratories) and 20 µM synthetic phospholipid but no Ca^2+^. For calibration, 20 µL of Thrombin Calibrator (Thrombinoscope) was added instead of the triggering solution. 20 µL of FluCa-reagent prepared from FluCa-kit (Thrombinoscope) was dispensed to initiate the reaction. The thrombograms and peak height were analyzed by the software. Data were collected in triplicate.

### Pharmacokinetic Study of hBS910 in Cynomolgus Monkeys

A single dose of 0.3 mg/kg of hBS910 was intravenously or subcutaneously administered to male cynomolgus monkeys (*n* = 3 for each group). Blood samples were collected at an appropriate time after each administration. Plasma concentration of bispecific antibodies was determined by human IgG-specific ELISA. Pharmacokinetic parameters were calculated by WinNonlin Professional software (Pharsight).

### Kinetic Analysis of Bispecific Antibodies Binding to FIXa and FX Using Surface Plasmon Resonance

Kinetic analysis of bispecific antibodies was performed by surface plasmon resonance (SPR) using a Biacore T200 system (GE Healthcare). MabSelect SuRe Ligand (Recombinant Protein A; GE Healthcare) was immobilized onto a CM4 sensor chip (GE Healthcare). Then, anti-FIXa or anti-FX monospecific antibodies were injected into flow cell 2 to be captured. Natalizumab (Biogen-Idec Inc.) as a control human IgG_4_ antibody was also injected into flow cell 1 to be captured. Then, 0, 80, 160, 320, 640, or 1,280 nM human FIXa or FX dissolved in running buffer (10 mM HEPES [pH 7.4], 150 mM NaCl, 0.05% surfactant P20, 2.5 mM CaCl_2_) was injected at a flow rate of 30 µL/min to monitor the association phase for 120 s and the dissociation phase for 30 s.

## Supporting Information

Figure S1 **Generation of anti-FIXa/FX bispecific antibodies.** Heavy chain variable regions (V_H_) of anti-FIXa or anti-FX antibodies were fused with engineered human IgG_2_ or IgG_4_ constant region having mutations to facilitate Fc heterodimerization. Light chain variable regions (V_L_) were fused with human κ or λ constant region. Bispecific antibodies were generated by expression with two pairs of genes, anti-FIXa and anti-FX heavy chain and light chain genes (or a common light chain gene).
(PDF)

Figure S2 **FR/CDR shuffling of the light chain.** (A) CDRs of three light chains (c1L, c2L and c3L) were shuffled among each other and grafted onto the FRs of c1L and c3L. Each light chain variant was expressed with the selected anti-FIXa and anti-FX heavy chains. (B) Effects of bispecific antibodies (67 nM) with light chain variants on APTT assay in FVIII-deficient plasma are shown. The Y-axis indicates the APTT (s). All the data were collected in duplicate and are expressed as mean.
(PDF)

Figure S3 **Effect of humanization of the lead chimeric antibody (BS15) on FVIII-mimetic activity.** Effect of chimeric antibody BS15 (circles) or humanized antibody hBS1 (squares) on FX activation in the presence of FIXa, FX, and synthetic phospholipid. The Y-axis indicates the 405 nm absorbance at 120 min of chromogenic development in the chromogenic substrate assay. All the data were collected in triplicate and are expressed as mean ± s.d (in many cases, the bars depicting s.d. are shorter than the height of the symbols).
(PDF)

Figure S4 **Positive charge cluster and Tyr30Glu mutation on anti-FIXa Fv of hBS106.** The positive charge cluster consists of arginine or lysine residues at Kabat position 60, 61, and 95 in the heavy chain and Kabat positions 24, 27, 31, 53, 54, 61, and 66 in the light chain of hBS106. Tyrosine located at Kabat position 30 in the light chain was mutated to glutamic acid to neutralize the positive charge cluster. Blue, red and gray colored surface indicates positively charged, negatively charged and neutral protein surface, respectively. Red and green line indicates heavy and light chain, respectively.
(PDF)

Figure S5 **Precipitation and liquid–liquid phase separation of bispecific antibody solution.** Micro CCD camera images of states of bispecific antibody solution showing a clear solution, precipitation, and liquid–liquid phase separation.
(PDF)

Figure S6 ***In silico*** **prediction of immunogenicity of bispecific antibodies.** (A) Immunogenicity risk score of BS15, hBS1, hBS910, trastuzumab, and palivizumab predicted by Epibase. (B) Immunogenicity scale of hBS910 and other marketed monoclonal antibodies by EpiMatrix. In both prediction systems, higher score indicates higher risk of immunogenicity in human.
(PDF)

Figure S7 **Necessity of bispecific binding to FIXa and FX for FVIII-mimetic activity.** Effect of the bispecific antibody (hBS910) (circles), monospecific anti-FIXa antibody (squares), monospecific anti-FX antibody (triangles), or a mixture of the two monospecific antibodies (diamonds) on FX activation in the presence of FIXa, FX, and synthetic phospholipid. The Y-axis indicates the 405 nm absorbance at 30 min of chromogenic development in the chromogenic substrate assay. All the data were collected in triplicate and are expressed as mean ± s.d (in many cases, the bars depicting s.d. are shorter than the height of the symbols). Monospecific antibodies against FIXa or FX antibodies or the mixture of them did not exhibit any detectable activity even at 120 min of chromogenic development.
(PDF)

Figure S8 **Surface plasmon resonance analysis of bispecific antibodies binding to FIXa and FX.** Sensorgrams of hBS1, hBS106, and hBS910 binding to FIXa (A) and FX (B) at a concentration of 80 nM, 160 nM, 320 nM, 640 nM, and 1280 nM.
(PDF)

Table S1 (PDF)

Table S2 (PDF)

Table S3 (PDF)
