# Supplementary figures and images for: Identification and Multidimensional Optimization of an Asymmetric Bispecific IgG Antibody Mimicking the Function of Factor VIII Cofactor Activity
Source: PLoS One. 2013 Feb 28;8(2):e57479. doi: 10.1371/journal.pone.0057479 (PMC3585358; doi:10.1371/journal.pone.0057479)

# Supplementary Figure S1

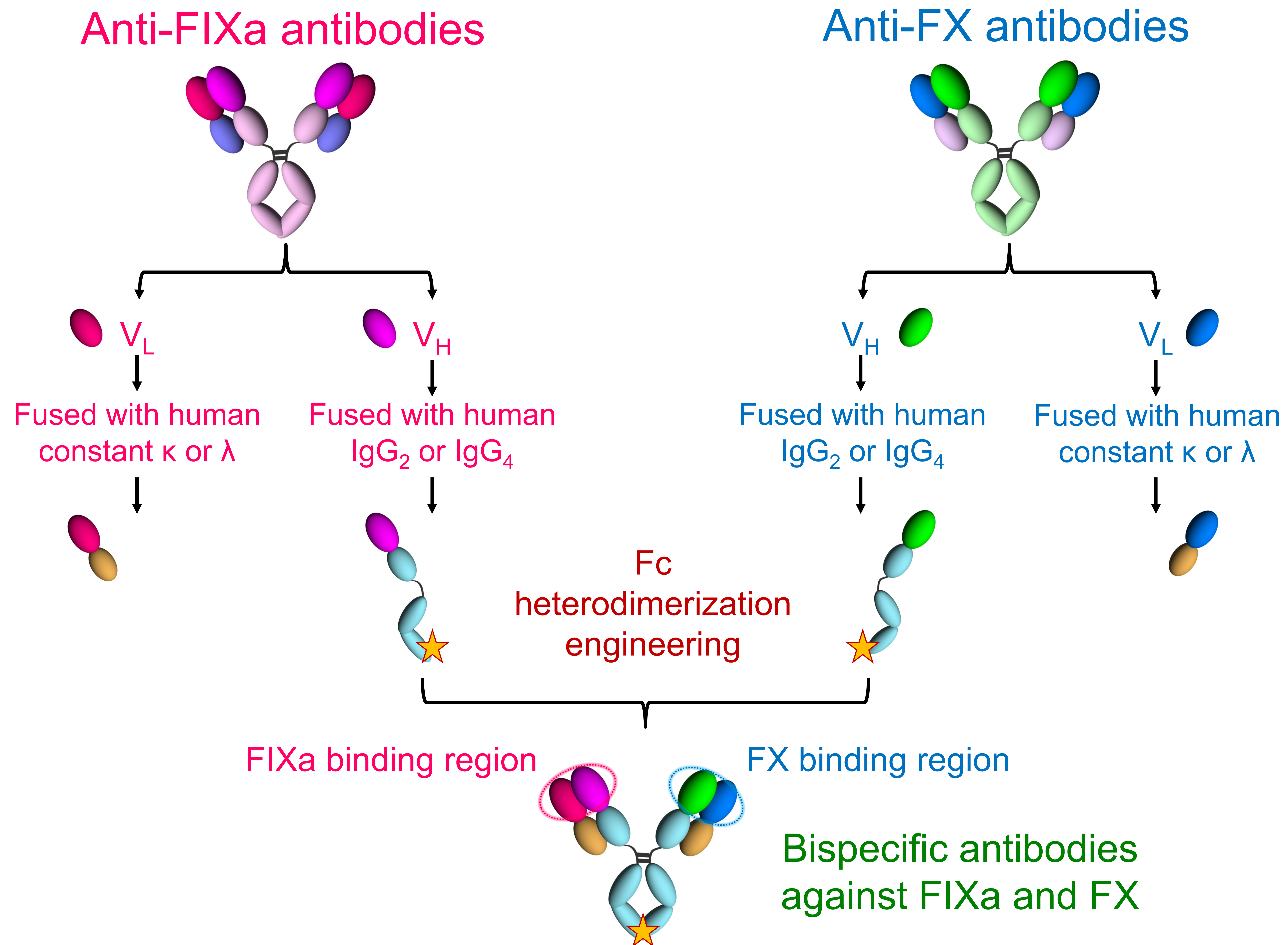

Supplement: Figure S1 — Generation of anti-FIXa/FX bispecific antibodies. Heavy chain variable regions (VH) of anti-FIXa or anti-FX antibodies were fused with engineered human IgG2 or IgG4 constant region having mutations to facilitate Fc heterodimerization. Light chain variable regions (VL) were fused with human κ or λ constant region. Bispecific antibodies were generated by expression with two pairs of genes, anti-FIXa and anti-FX heavy chain and light chain genes (or a common light chain gene). (PDF) [file pone.0057479.s001.pdf]

Supplementary Figure S3

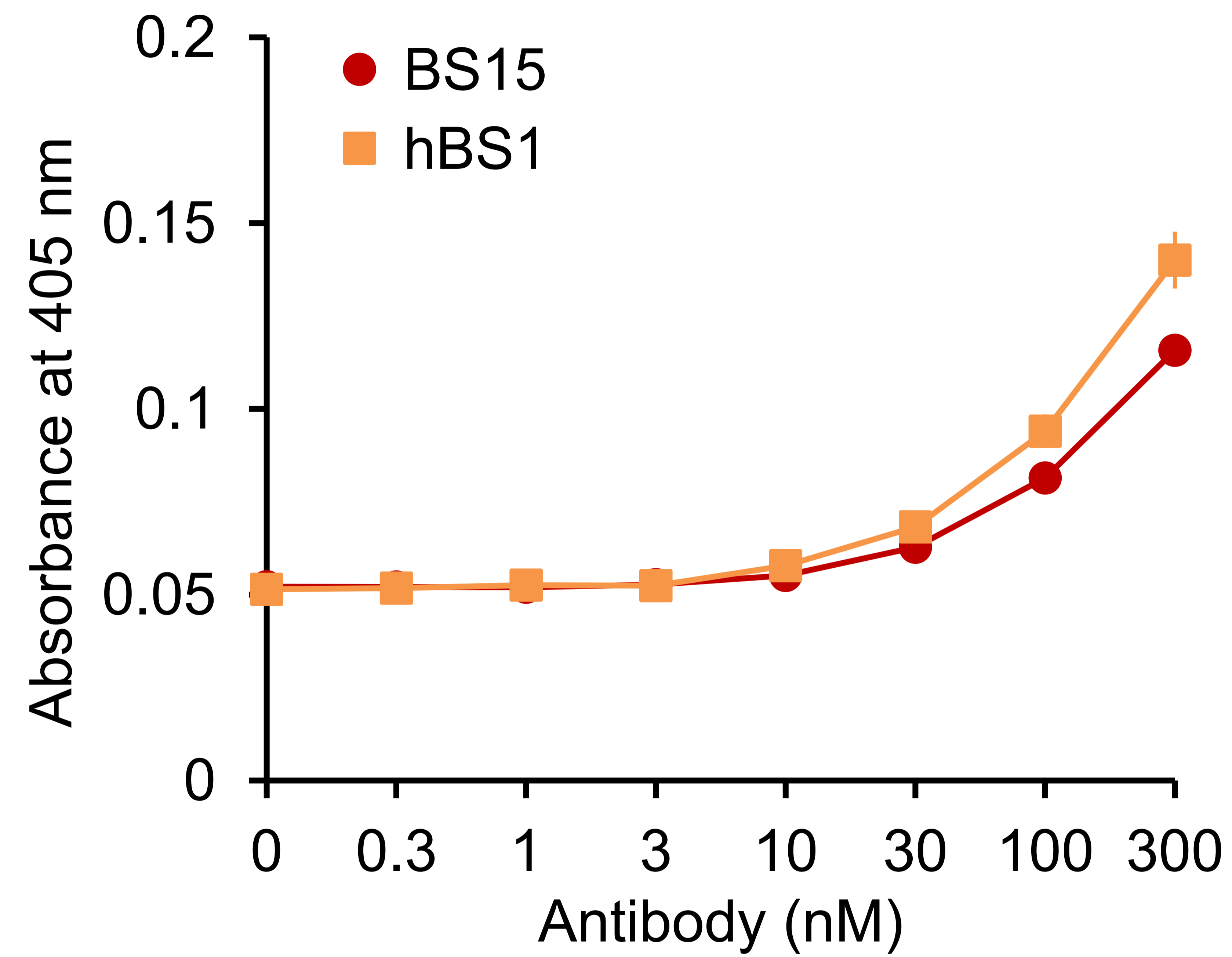

Supplement: Figure S3 — Effect of humanization of the lead chimeric antibody (BS15) on FVIII-mimetic activity. Effect of chimeric antibody BS15 (circles) or humanized antibody hBS1 (squares) on FX activation in the presence of FIXa, FX, and synthetic phospholipid. The Y-axis indicates the 405 nm absorbance at 120 min of chromogenic development in the chromogenic substrate assay. All the data were collected in triplicate and are expressed as mean ± s.d (in many cases, the bars depicting s.d. are shorter than the height of the symbols). (PDF) [file pone.0057479.s003.pdf]

Supplementary Figure S4

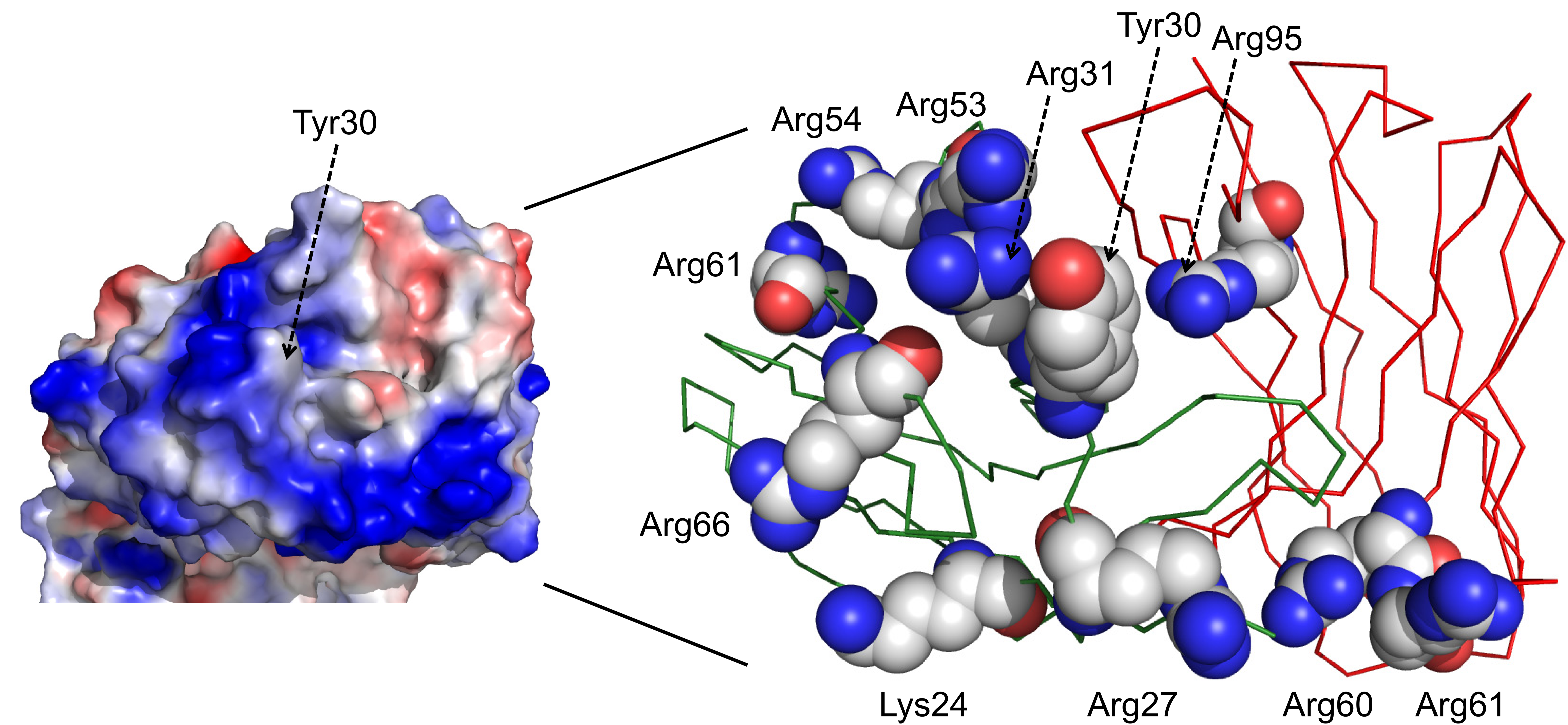

Supplement: Figure S4 — Positive charge cluster and Tyr30Glu mutation on anti-FIXa Fv of hBS106. The positive charge cluster consists of arginine or lysine residues at Kabat position 60, 61, and 95 in the heavy chain and Kabat positions 24, 27, 31, 53, 54, 61, and 66 in the light chain of hBS106. Tyrosine located at Kabat position 30 in the light chain was mutated to glutamic acid to neutralize the positive charge cluster. Blue, red and gray colored surface indicates positively charged, negatively charged and neutral protein surface, respectively. Red and green line indicates heavy and light chain, respectively. (PDF) [file pone.0057479.s004.pdf]

# Supplementary Figure S5

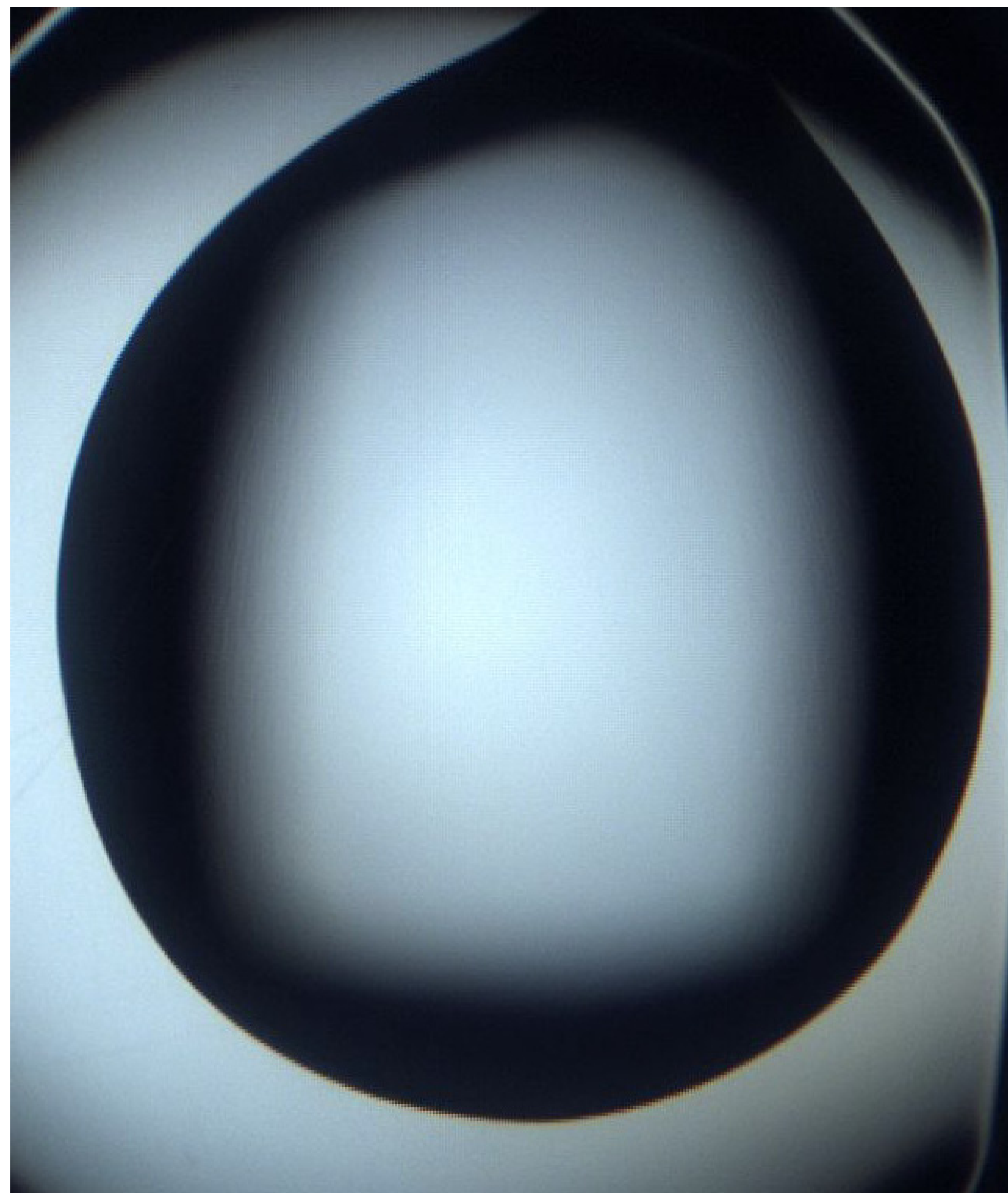

Clear solution

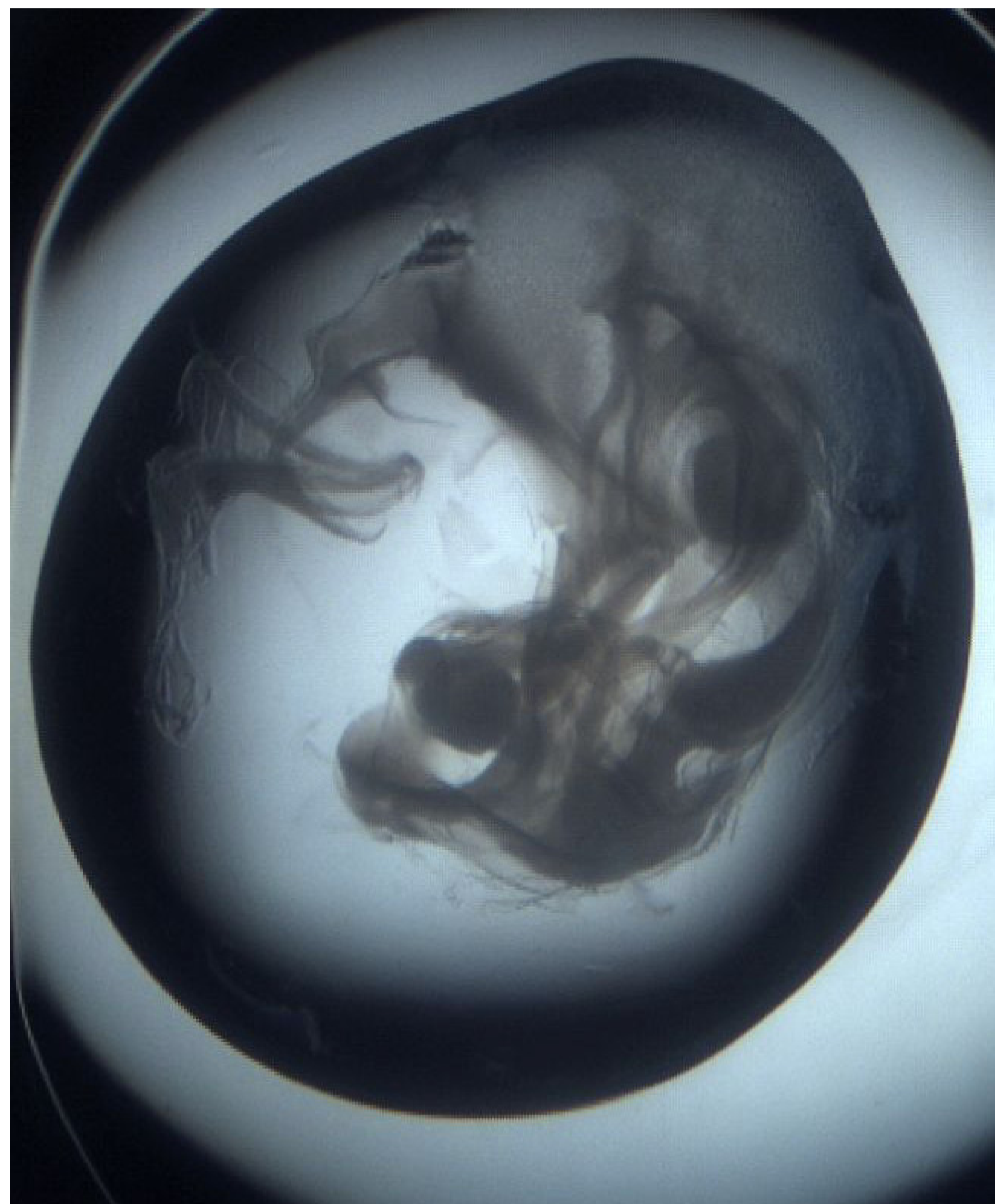

Precipitation

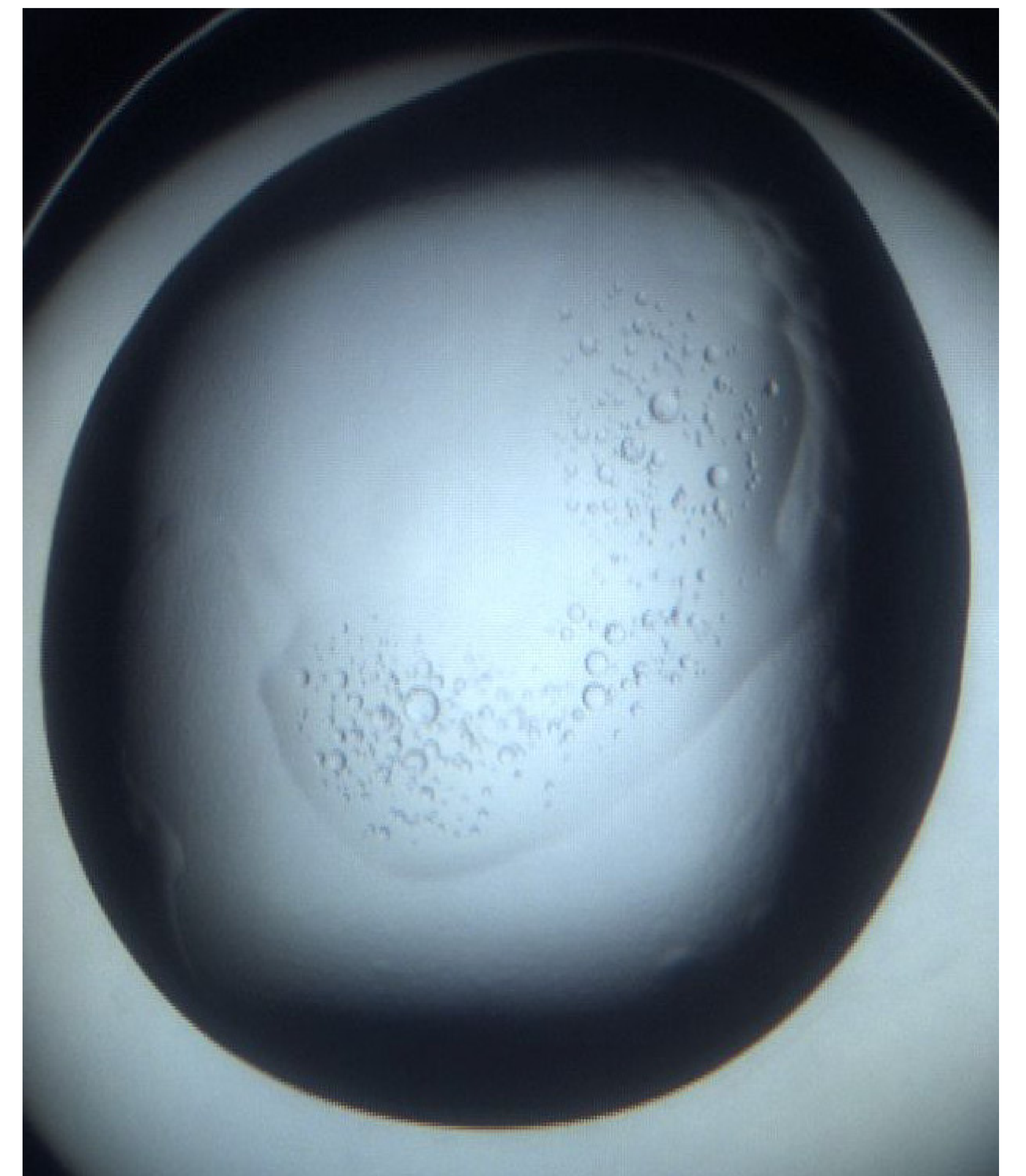

Liquid–liquid phase  
separation

Supplement: Figure S5 — Precipitation and liquid–liquid phase separation of bispecific antibody solution. Micro CCD camera images of states of bispecific antibody solution showing a clear solution, precipitation, and liquid–liquid phase separation. (PDF) [file pone.0057479.s005.pdf]

# Supplementary Figure S6

**A**

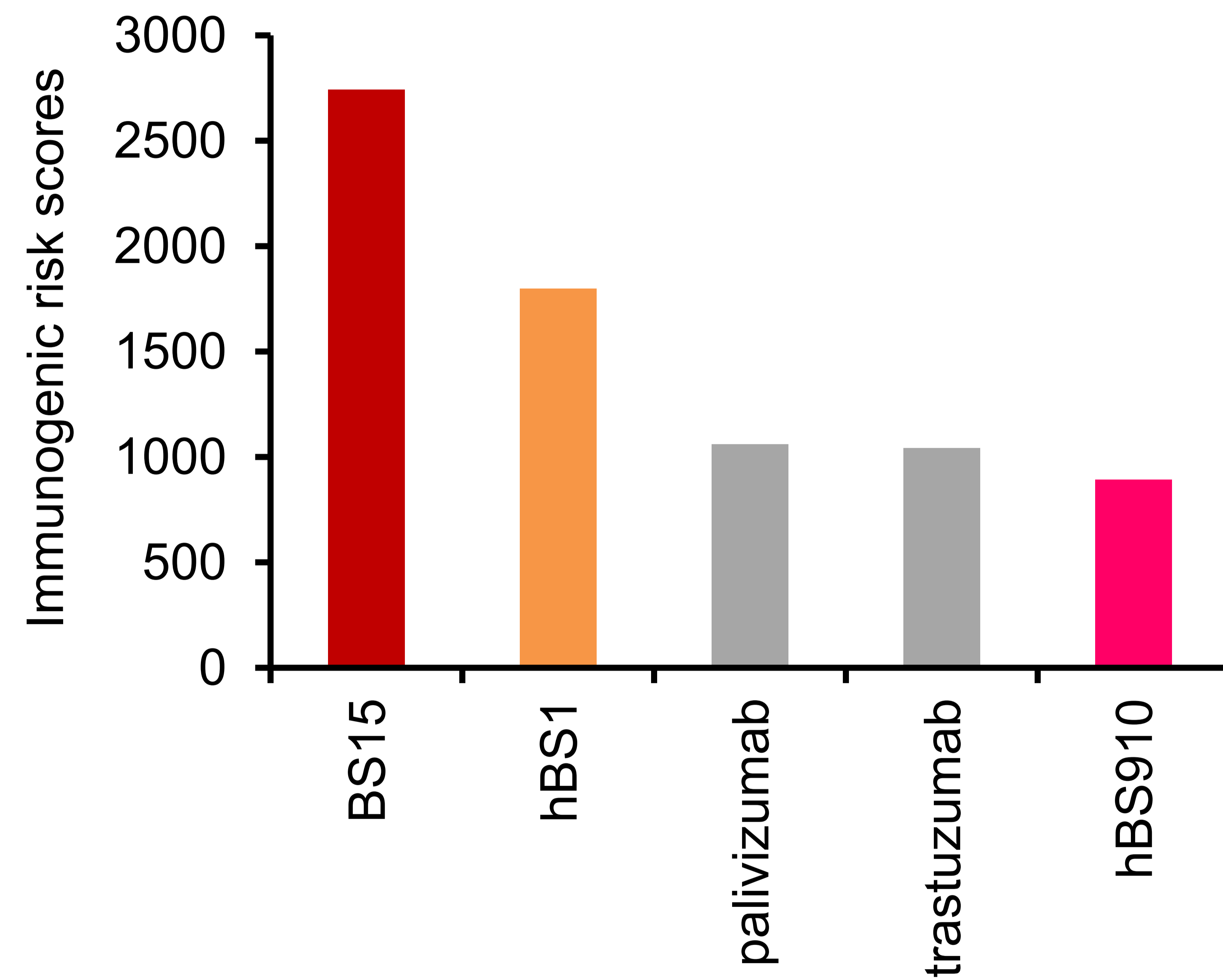

**B**

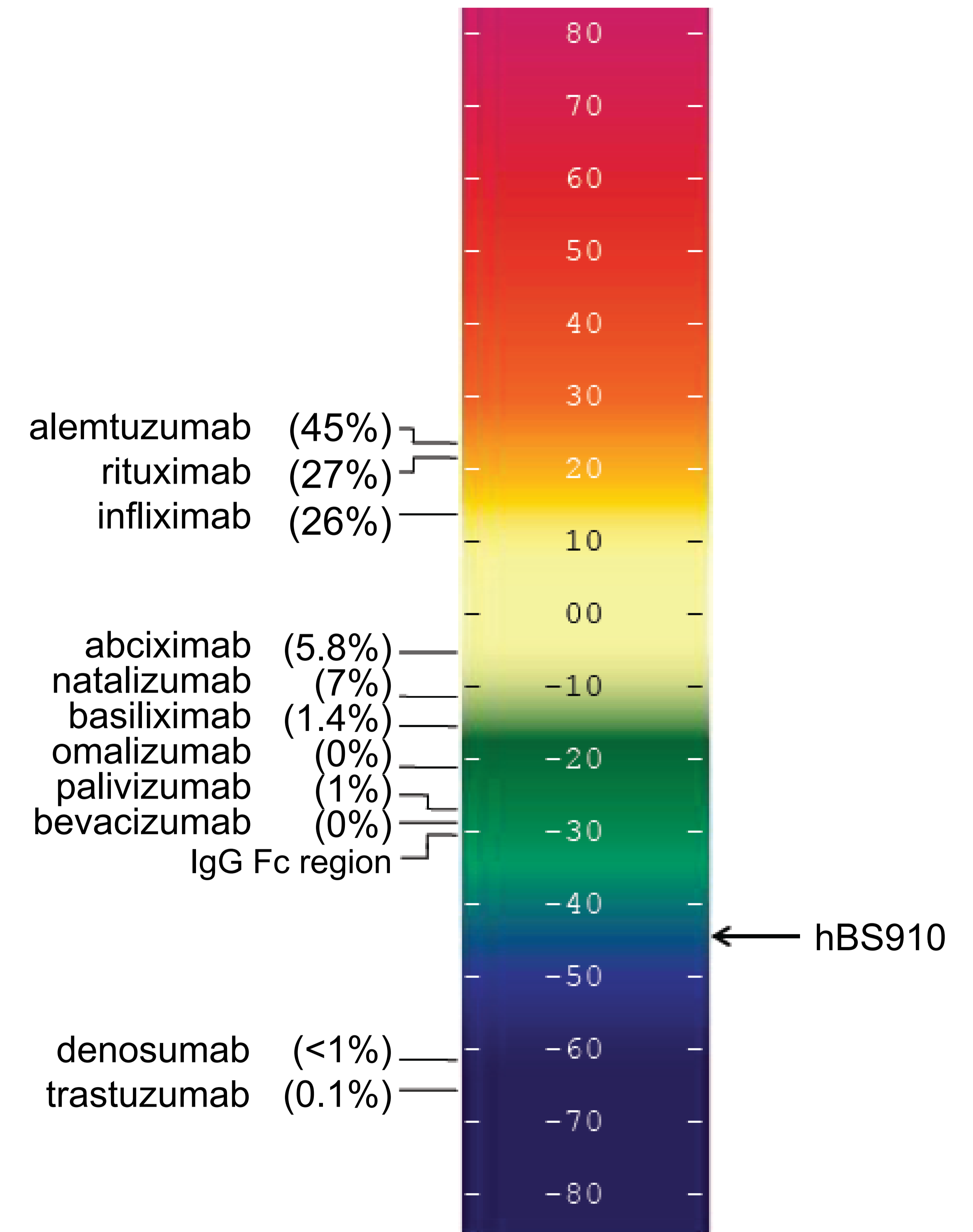

Supplement: Figure S6 — In silico prediction of immunogenicity of bispecific antibodies. (A) Immunogenicity risk score of BS15, hBS1, hBS910, trastuzumab, and palivizumab predicted by Epibase. (B) Immunogenicity scale of hBS910 and other marketed monoclonal antibodies by EpiMatrix. In both prediction systems, higher score indicates higher risk of immunogenicity in human. (PDF) [file pone.0057479.s006.pdf]

# Supplementary Figure S7

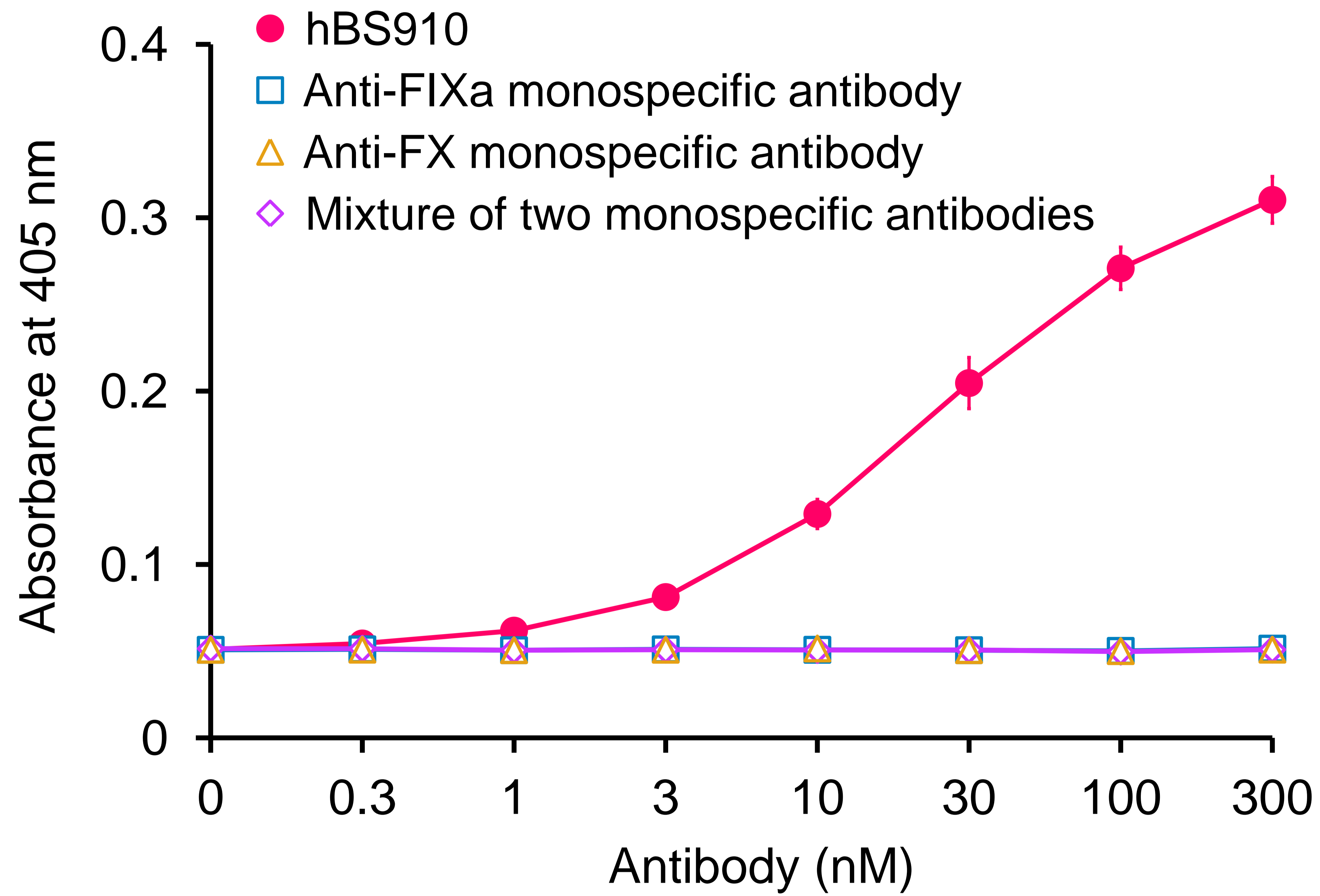

Supplement: Figure S7 — Necessity of bispecific binding to FIXa and FX for FVIII-mimetic activity. Effect of the bispecific antibody (hBS910) (circles), monospecific anti-FIXa antibody (squares), monospecific anti-FX antibody (triangles), or a mixture of the two monospecific antibodies (diamonds) on FX activation in the presence of FIXa, FX, and synthetic phospholipid. The Y-axis indicates the 405 nm absorbance at 30 min of chromogenic development in the chromogenic substrate assay. All the data were collected in triplicate and are expressed as mean ± s.d (in many cases, the bars depicting s.d. are shorter than the height of the symbols). Monospecific antibodies against FIXa or FX antibodies or the mixture of them did not exhibit any detectable activity even at 120 min of chromogenic development. (PDF) [file pone.0057479.s007.pdf]

# Supplementary Figure S8

**A**

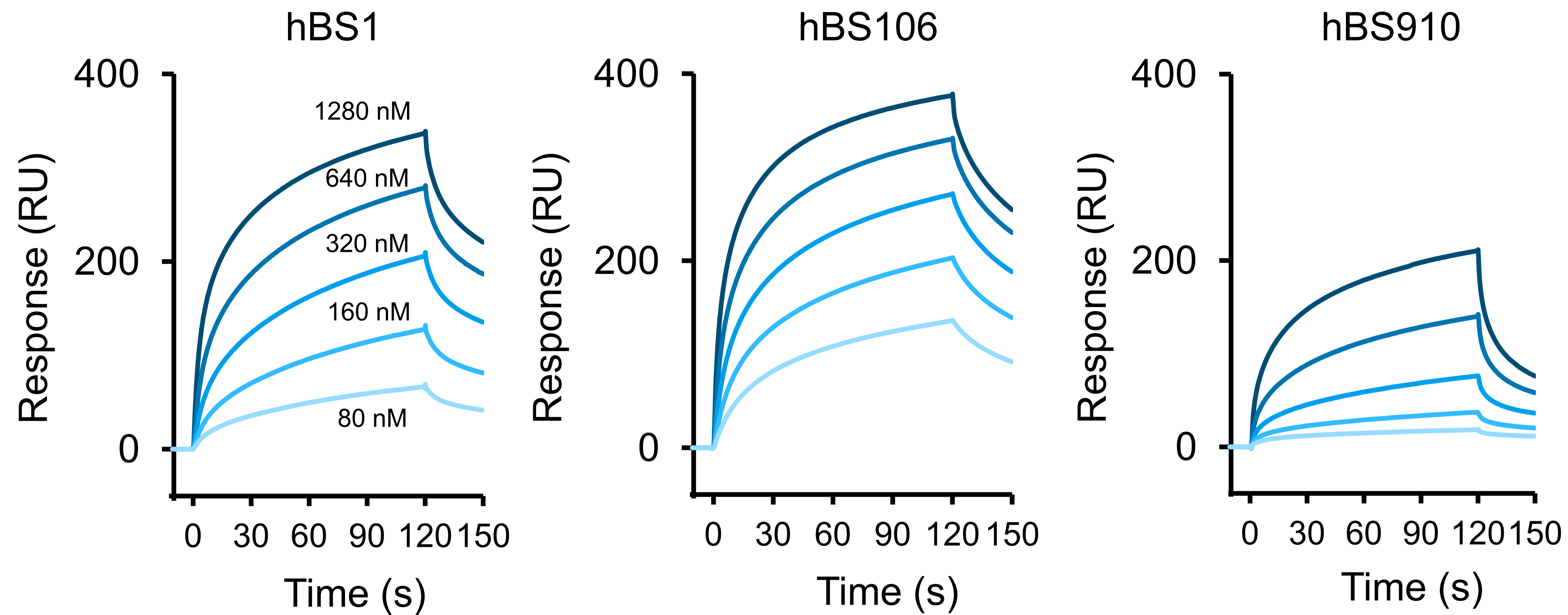

**B**

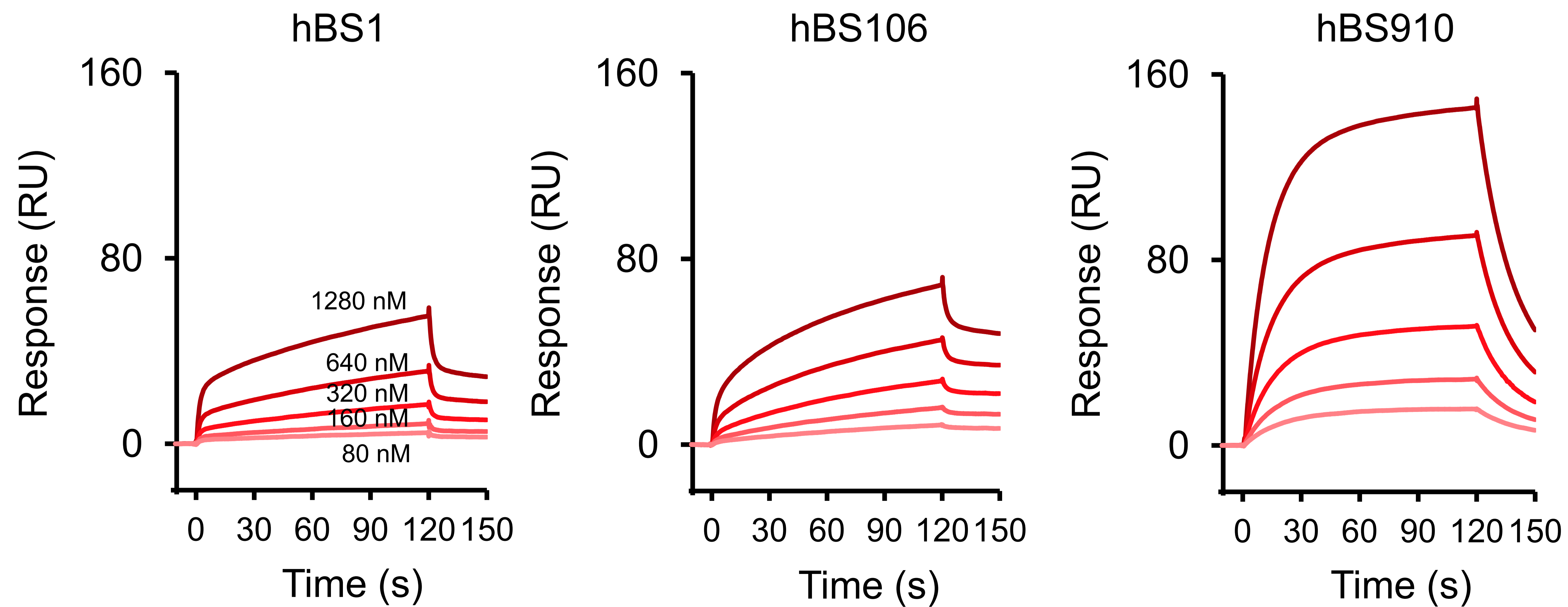

Supplement: Figure S8 — Surface plasmon resonance analysis of bispecific antibodies binding to FIXa and FX. Sensorgrams of hBS1, hBS106, and hBS910 binding to FIXa (A) and FX (B) at a concentration of 80 nM, 160 nM, 320 nM, 640 nM, and 1280 nM. (PDF) [file pone.0057479.s008.pdf]
