# Supplementary material for: Identification and Multidimensional Optimization of an Asymmetric Bispecific IgG Antibody Mimicking the Function of Factor VIII Cofactor Activity
Source: PLoS One. 2013 Feb 28;8(2):e57479. doi: 10.1371/journal.pone.0057479 (PMC3585358; doi:10.1371/journal.pone.0057479)
Supplement: Figure S2 — FR/CDR shuffling of the light chain. (A) CDRs of three light chains (c1L, c2L and c3L) were shuffled among each other and grafted onto the FRs of c1L and c3L. Each light chain variant was expressed with the selected anti-FIXa and anti-FX heavy chains. (B) Effects of bispecific antibodies (67 nM) with light chain variants on APTT assay in FVIII-deficient plasma are shown. The Y-axis indicates the APTT (s). All the data were collected in duplicate and are expressed as mean. (PDF) [file pone.0057479.s002.pdf]

Supplementary Figure S2

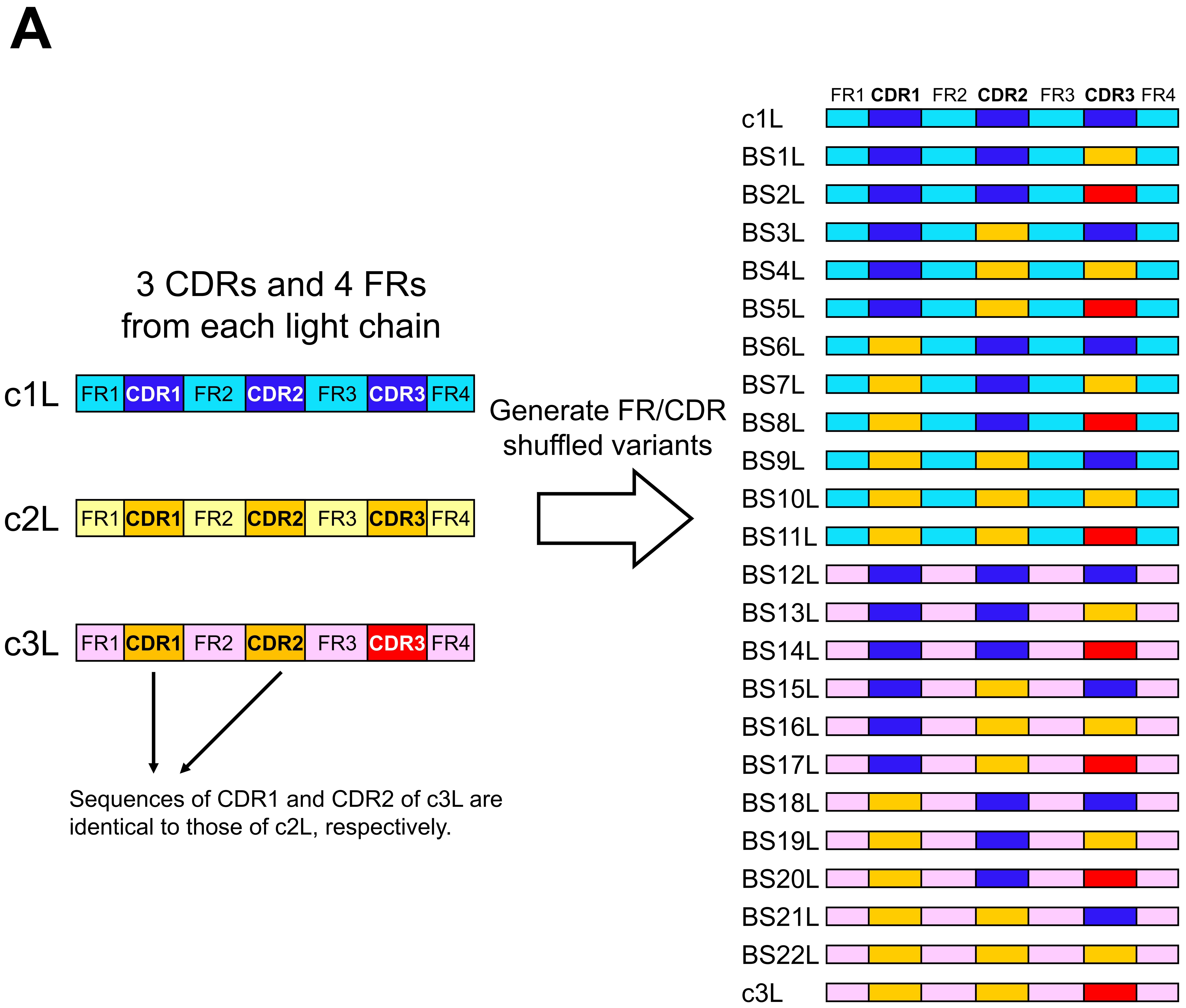

B

	APTT (s)
(-)	85.3
c1L	37.1
BS1L	56.7
BS2L	36.6
BS3L	33.1
BS4L	54.7
BS5L	36.3
BS6L	76.3
BS7L	79.5
BS8L	75.8
BS9L	57.0
BS10L	82.0
BS11L	68.5
BS12L	25.6
BS13L	28.8
BS14L	25.8
BS15L	23.4
BS16L	29.0
BS17L	25.6
BS18L	46.0
BS19L	56.4
BS20L	51.2
BS21L	31.8
BS22L	44.2
c3L	36.3
