# Supplementary material for: Identification and Multidimensional Optimization of an Asymmetric Bispecific IgG Antibody Mimicking the Function of Factor VIII Cofactor Activity
Source: PLoS One. 2013 Feb 28;8(2):e57479. doi: 10.1371/journal.pone.0057479 (PMC3585358; doi:10.1371/journal.pone.0057479)
Supplement: Table S1 — (PDF) [file pone.0057479.t001.pdf]

Supplementary Table S1

Supplementary Table S1. Number of mutations from hBS1 (variable region)

	Anti-FIXa heavy chain	Anti-FX heavy chain	Common light chain	Total
hBS23	7	14	14	35
hBS106	2	2	9	13
hBS128	2	2	10	14
hBS228	8	9	10	27
hBS366	2	9	10	21
hBS376	8	2	10	20
hBS560	13	17	12	42
hBS660	15	17	12	44
hBS910	15	17	14	46
