# Supplementary material for: Identification and Multidimensional Optimization of an Asymmetric Bispecific IgG Antibody Mimicking the Function of Factor VIII Cofactor Activity
Source: PLoS One. 2013 Feb 28;8(2):e57479. doi: 10.1371/journal.pone.0057479 (PMC3585358; doi:10.1371/journal.pone.0057479)
Supplement: Table S2 — (PDF) [file pone.0057479.t002.pdf]

Supplementary Table S2

Supplementary Table S2. Pharmacokinetic parameters of hBS106, hBS128, and hBS228 in mice after subcutaneous injection

	$T_{1/2}$ (day)	C_{max} (μg/mL)	AUC (μg・day/mL)	CL/F (mL/day/kg)
hBS106	5.19 ± 0.95	1.62 ± 0.16	14.96 ± 0.26	66.86 ± 1.18
hBS128	7.27 ± 1.79	5.54 ± 0.39	63.13 ± 6.00	15.94 ± 1.56
hBS228	5.42 ± 2.47	9.15 ± 0.65	125.33 ± 15.66	8.07 ± 1.04
