# Supplementary material for: Identification and Multidimensional Optimization of an Asymmetric Bispecific IgG Antibody Mimicking the Function of Factor VIII Cofactor Activity
Source: PLoS One. 2013 Feb 28;8(2):e57479. doi: 10.1371/journal.pone.0057479 (PMC3585358; doi:10.1371/journal.pone.0057479)
Supplement: Table S3 — (PDF) [file pone.0057479.t003.pdf]

Supplementary Table S3

Supplementary Table S3. Pharmacokinetic parameters of hBS910 in cynomolgus monkeys

	$T_{1/2}$ (day)	C_{max} (μg/mL)	AUC_{last} (μg・day/mL)	AUC_{INF} (μg・day/mL)	CL or CL/F (mL/day/kg)	F
intravenous	23.0 ± 1.77		75.5 ± 4.07	90.4 ± 5.27	3.32 ± 0.19	
subcutaneous	19.2 ± 0.60	2.64 ± 0.02	67.1 ± 7.49	78.0 ± 10.79	3.89 ± 0.54	0.86 ± 0.12
